# Identification of hub genes and construction of diagnostic nomogram model in schizophrenia

**DOI:** 10.3389/fnagi.2022.1032917

**Published:** 2022-10-14

**Authors:** Chi Zhang, Naifu Dong, Shihan Xu, Haichun Ma, Min Cheng

**Affiliations:** ^1^Department of Anesthesiology, The First Hospital of Jilin University, Changchun, China; ^2^College of Basic Medical Sciences, Jilin University, Changchun, China

**Keywords:** schizophrenia, biomarker, neuroimmune, diagnosis, nomogram, drug prediction

## Abstract

Schizophrenia (SCZ), which is characterized by debilitating neuropsychiatric disorders with significant cognitive impairment, remains an etiological and therapeutic challenge. Using transcriptomic profile analysis, disease-related biomarkers linked with SCZ have been identified, and clinical outcomes can also be predicted. This study aimed to discover diagnostic hub genes and investigate their possible involvement in SCZ immunopathology. The Gene Expression Omnibus (GEO) database was utilized to get SCZ Gene expression data. Differentially expressed genes (DEGs) were identified and enriched by Gene Ontology (GO), Kyoto Encyclopedia of Genes and Genomes (KEGG), and disease ontology (DO) analysis. The related gene modules were then examined using integrated weighted gene co-expression network analysis. Single-sample gene set enrichment (GSEA) was exploited to detect immune infiltration. SVM-REF, random forest, and least absolute shrinkage and selection operator (LASSO) algorithms were used to identify hub genes. A diagnostic model of nomogram was constructed for SCZ prediction based on the hub genes. The clinical utility of nomogram prediction was evaluated, and the diagnostic utility of hub genes was validated. mRNA levels of the candidate genes in SCZ rat model were determined. Finally, 24 DEGs were discovered, the majority of which were enriched in biological pathways and activities. Four hub genes (NEUROD6, NMU, PVALB, and NECAB1) were identified. A difference in immune infiltration was identified between SCZ and normal groups, and immune cells were shown to potentially interact with hub genes. The hub gene model for the two datasets was verified, showing good discrimination of the nomogram. Calibration curves demonstrated valid concordance between predicted and practical probabilities, and the nomogram was verified to be clinically useful. According to our research, NEUROD6, NMU, PVALB, and NECAB1 are prospective biomarkers in SCZ and that a reliable nomogram based on hub genes could be helpful for SCZ risk prediction.

## Introduction

Schizophrenia (SCZ) is a multifaceted mental illness with a broad variety of clinical and physiological manifestations; this disorder affects 20 million people and ranks among the top 25 leading causes of disability worldwide (Roy, 1986; GBD 2019 Diseases and Injuries Collaborators, 2020). SCZ is related with an approximately 15-year reduction in life expectancy in comparison to the gross population and a 5–10 percent lifetime risk of suicide. The low quality of life caused by cognitive impairment and mortality risks make SCZ a severe public health burden (Cloutier et al., 2016; Avramopoulos, 2018; Wahbeh and Avramopoulos, 2021). Despite the abundance of literature of SCZ manifestations, its exact etiology and pathogenesis are poorly known. Therefore, research on the pathogenesis and genetic mechanisms of SCZ is crucial.

The whole-transcriptome gene expression profiling study has been extensively utilized to discover SCZ-associated genes, identify disease-associated biomarkers, and anticipate treatment benefit. FOS was found to be a biomarker related to central and peripheral changes in SCZ (Huang et al., 2019), with NFKBIA, CDKN1A, BTG2, and GADD45B being recognized as core SCZ genes (Feng et al., 2022). Autophagy-related competing endogenous RNAs have been found to exhibit diagnostic efficacy in SCZ (Li R. et al., 2021). Moreover, S100B is regarded as a marker of nervous system impairment, and elevated levels have been seen in individuals with SCZ at illness onset as well as in drug-naive patients (Langeh et al., 2021). Several additional immunological indicators in microglia cells, such as cyclooxygenase-2 (COX-2) and prostaglandin E2 (PGE2), have been proposed as possible new therapeutic targets for SCZ treatment (Najjar et al., 2013). However, due to a lack of objective diagnostic methods, definitive assessment and therapy selection for SCZ remain problematic. To increase the efficacy of treatment methods, it is critical to develop innovative biomarkers that are strongly connected with SCZ.

Our research intended to investigate gene expression alterations in the pathophysiology of SCZ and to develop new possible diagnostic biomarkers. In this work, we scrutinized two Gene Expression Omnibus (GEO) datasets and sorted out 24 differentially expressed genes (DEGs) from prefrontal cortex (PFC) samples. The essential modules associated with SCZ were identified and four hub genes, NEUROD6, NMU, PVALB, and NECAB1, were sorted using the support vector machine–recursive feature elimination (SVM-RFE), random forest (RF), and least absolute shrinkage and selection operator (LASSO) algorithms. Then, utilizing hub genes, we developed and validated a predictive nomogram for clinical SCZ diagnosis. The diagnostic values of the four hub genes and the nomogram model were validated with good accuracy per receiver operating characteristics (ROC) curves. The selected four hub genes and nomogram could help improve SCZ diagnosis in high-risk patients, thereby helping to elucidate the neuropsychiatric etiology of SCZ.

## Materials and methods

### Data processing

**FIGURE 1:**
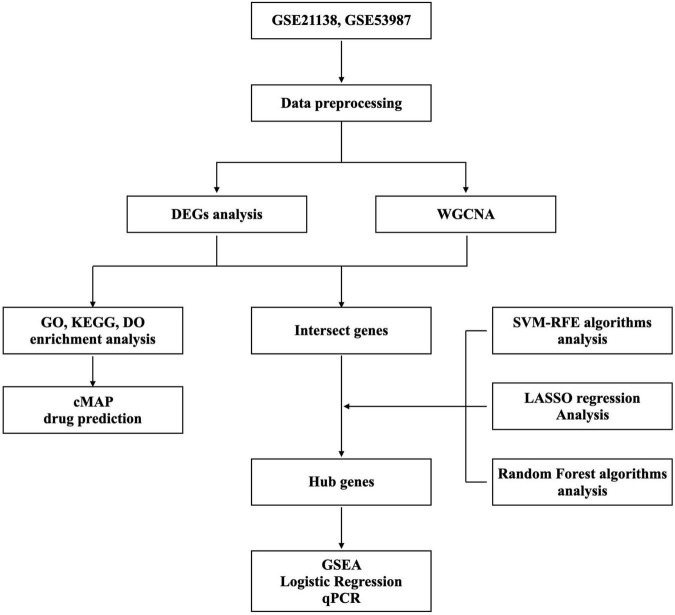
Study workflow.

GSE21138^1^ and GSE53987^2^ [GPL570 platform (HG-U133_Plus_2) Affymetrix Human Genome U133 Plus 2.0] were obtained from the GEO database. We collected datasets including PFC samples, of which GSE21138 contained 29 normal and 30 SCZ samples and GSE53987 contained 19 normal and 15 SCZ samples. Then, the R packages limma and sva were applied for profiles combination and the normalization. Probes not matching any known gene were eliminated. If more than one probe matched to a gene, the average expression was aggregated. The Perl programming language was used to remove lncRNA profiles and identify mRNA matrix files. The R package ggplot2 was used to normalize data after processing. Information of datasets is listed in Supplementary Table 1. The study’s flow diagram is shown in Figure 1.

### Differentially expressed genes identification

To find DEGs between SCZ and healthy samples, the limma R package was employed. The cutoff criteria were adjusted *P* < 0.05 and | log fold change (FC) | > 0.5. Using the ggplots package, the heatmap and volcano diagram were generated.

### Enrichment analysis

To determine the biological implications of genes and functions, DEGs were subjected to GO, Kyoto Encyclopedia of Genes and Genomes (KEGG), and DO analyses using clusterProfiler and DOSE package. A *P*-value of less than 0.05 was set as the cutoff criterion.

### Gene set enrichment

GSEA is a computer tool to determine the accordance of a highly enriched gene set. The reference gene set, “c2.cp.kegg.v6.2. symbols.gmt,” was downloaded from the Molecular Signature Database (MSigDB). Enrichment sets comprising fewer than 10 or more than 200 genes were omitted. The upregulated pathways had a normalized enrichment score (NES) greater than zero, whereas the downregulated pathways had a NES less than zero. Five of the most essential pathways were determined (FDR < 0.05).

### Weighted gene co-expression network analysis

We combined and batch-processed the data from GSE21138 and GSE53987. Weighted gene co-expression network analysis (WGCNA) package was used to assess the trait-related modules. A topological overlap matrix was constructed from the expression profile. The soft-thresholding power of 5 and minimum module size of 30 were set to screen core modules. A height limit of 0.25 was used as a guideline for modules combination. The modules were then tested using Pearson’s correlation test at a significance threshold of *P* 0.05.

### Support vector machine, random forest, and least absolute shrinkage and selection operator model construction

First, candidate genes were found by crossing DEGs with genes of WGCNA hub module. Next, hub genes were classified by overlapping genes from the SVM-RFE method with the e1071 package (Noble, 2006), the RF algorithm with the randomForest R package (Paul et al., 2018), and the LASSO algorithm with glmnet package (Vasquez et al., 2016).

### Single sample gene set enrichment analysis

Single sample gene set enrichment analysis (ssGSEA), using the GSVA package, was performed to compare the infiltration of 28 immune cells within normal and SCZ samples (Hänzelmann et al., 2013). We identified 28 immunocytes: immature dendritic cells, type 1 T helper cells, activated CD4 + T cells, T follicular helper cells, activated dendritic cells, CD56 dim NK cells, central memory CD4 + T cells, effector memory CD4 + T cells, eosinophils, gamma delta T cells, activated CD8 + T cells, CD56 bright natural killer (NK) cells, mast cells, myeloid-derived suppressor cells, B cells, effector memory CD8 + T cells, monocytes, natural killer cells, natural killer T cells, macrophages, neutrophils, plasmacytoid dendritic cells, regulatory T cells, central memory CD8 + T cells, immature B cells, type 17 T helper cells, and type 2 T helper cells, memory B cells.

### Nomogram model construction

To forecast the incidence of SCZ, rms package was applied to develop the diagnostic nomogram model. “Points” denotes scores of the corresponding factor. Following that, the nomogram model’s predictive ability was evaluated using a calibration curve (Chen et al., 2019). Finally, the practical applicability of the model was assessed using decision curve analysis (DCA) (Vickers and Elkin, 2006). We used the pROC package (Wolbers et al., 2009) to conduct ROC curve and the diagnostic capacities of hub genes and the nomogram model were examined using the area under the curve (AUC).

### qRT-PCR validation

SCZ models were obtained from rats injected intraperitoneally with saline or MK801 (Sigma-Aldrich, St.) (0.5 mg/kg body weight) for 6 days continuously. Total RNA from the rat PFCs was extracted with TRIzol reagent (Takara, Shiga, Japan). 500 ng mRNA in total was transcribed reversely using a Prime-Script RT reagent Kit (Takara), and qRT-PCR was performed at a final volume of 20 μL. Thermal settings were 95^°^C for 30 s, 40 cycles of 95^°^C for 10 s, and 60^°^C for 30 s. Hub gene expression was determined using the 2^–Δ^ ^Δ^ ^CT^ methodology. Primers information is shown in Table 1.

**TABLE 1:** Primer sequences used in this study.

Primers	NEUROD6
Forward	TCTAGAGGCTCCAGGAGAC
Reverse	GACTCGTCAAACGGTAGTG
Primers	NMU
Forward	CAAAGTGAATGAATACCAGGGTC
Reverse	GTTGACCTCTTCCCATTGC
Primers	PVALB
Forward	GCTAAGGAAACAAAGACGCT
Reverse	CAGAGTGGAGAATTCTTCAACC
Primers	NECAB1
Forward	AACTCCTCAGAAGAGCTCAG
Reverse	GTCTGCTCTCCTCAGTATGTC
Primers	GAPDH
Forward	AACTCCCATTCTTCCACCT
Reverse	TTGTCATACCAGGAAATGAGC

### Connectivity map analysis

The online platform Connectivity Map (CMap)^3^ was used to measure the connectivity between illnesses gene expression features and compound-induced gene signatures to better comprehend drug mechanisms and uncover novel therapeutic compounds. Thus, DEGs were uploaded to the CMap database to anticipate the possible therapeutic small-molecule medicines on SCZ.

### Statistical analysis

R software (version 4.1.3) was used for data examination. The Wilcoxon test was performed for groups comparation, and *P* < 0.05 was defined as a significant difference.

## Results

### Differentially expressed genes identification in schizophrenia and healthy control groups

**FIGURE 2:**
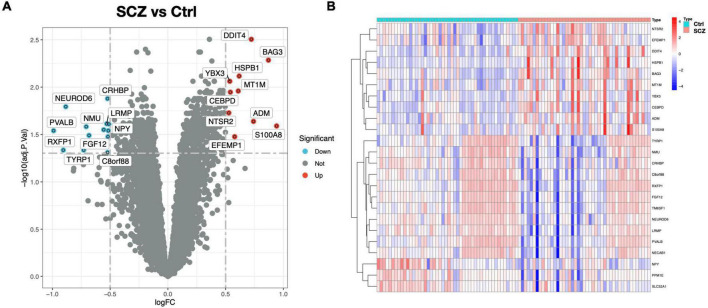
DEG screening between SCZ and healthy control. **(A)** Volcano graphic visualizing DEGs of SCZ and normal samples. **(B)** Heatmap of DEGs among normal and SCZ samples.

In this study, two microarray datasets (GSE21138 and GSE53987) were used to analyze differential expression. The expression matrix before and after normalization is shown in Supplementary Figure 1. In the integrated expression matrix, there were 24 DEGs revealed, with 10 upregulated and 14 downregulated, as shown in Figures 2A,B. The protein–protein interactions of the DEGs are shown in Supplementary Figure 2.

### Functional analysis

**FIGURE 3:**
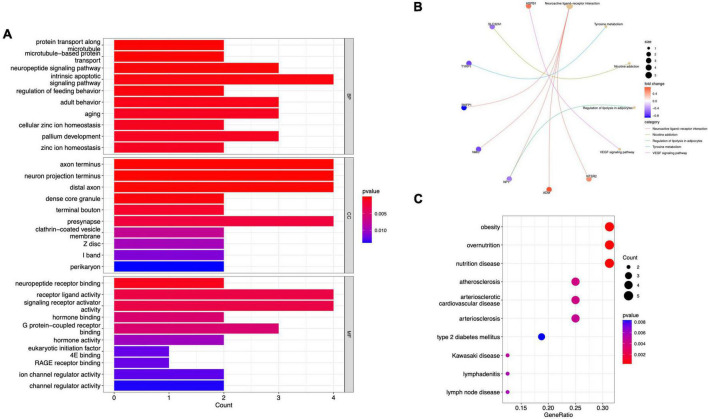
Functional DEG enrichment. **(A)** GO analysis. **(B)** KEGG pathway analysis. **(C)** DO analysis.

**FIGURE 4:**
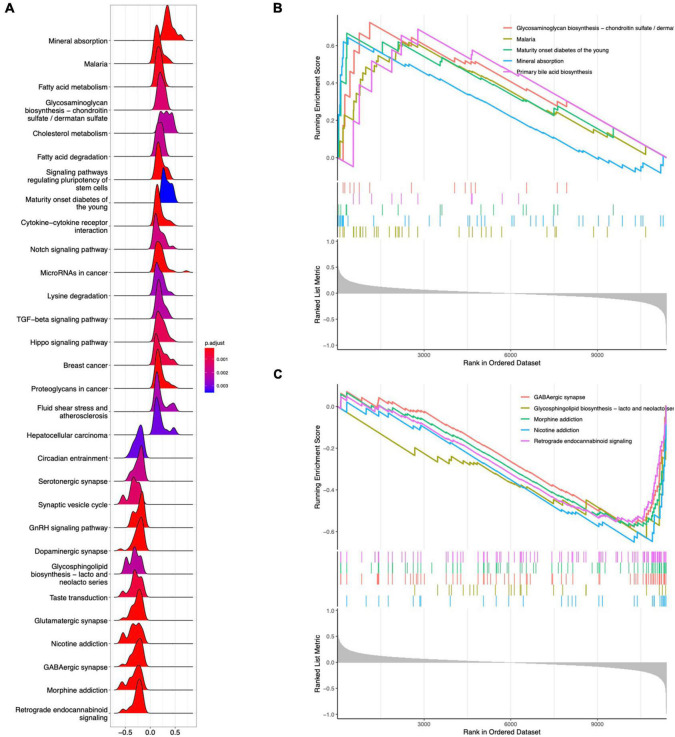
GSEA analysis for DEGs. **(A)** Ridgeline plot of GSEA analysis results. **(B)** Top five enrichment terms for upregulated DEGs. **(C)** Top five enrichment terms for downregulated DEGs.

GO analysis revealed 232 biological processes (BP), 29 cellular components (CC), and 28 molecular functions (MF), as shown in Supplementary Table 2. Figure 3A lists the top 10 GO items. DEGs were significantly enriched in neuropeptide pathways, adult behavior, aging, pallium development, axon terminals, neuron projection terminals, and receptor-ligand activity. According to KEGG analysis, DEGs were enriched in neurofunctional ligand-receptor interactions, as shown in Figure 3B. DO analysis revealed 54 items, as shown in Supplementary Table 3. Figure 3C shows the top ten items revealed by each functional and enrichment analysis. GSEA as shown in Supplementary Table 4 demonstrated the genes upregulated were primarily enriched in the Notch and TGF-beta signaling pathway, as shown in Figures 4A,B; downregulated genes were enriched in neurofunctional processes, as shown in Figure 4A, including GABAergic synapses, serotonergic synapses, circadian entrainment, synaptic vesicle cycle, morphine addiction, and dopaminergic synapses. Figure 4C shows the top five items.

### Overlap between schizophrenia-related module genes with differentially expressed genes

**FIGURE 5:**
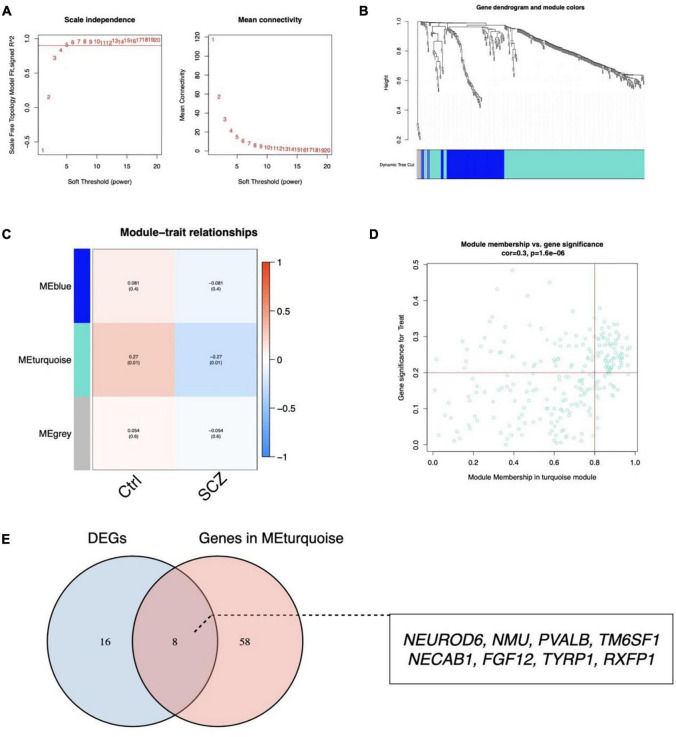
Identification of critical modules by WGCNA. **(A)** Scale-free fit index and mean connectivity for different soft-thresholding powers. **(B)** Topological overlap dissimilarity aggregation of DEGs clusters. **(C)** Module-feature correlations Each row represents a module list, whereas each column represents a clinical characteristic. The first line of each cell includes the associated correlation, while the second line gives the *P*-value. **(D)** Scatter plot of the turquoise module. **(E)** Venn diagram for overlapped genes.

A scale-free network with a soft threshold of 5 (*R*^2^ = 0.91) was built, as shown in Figure 5A and Supplementary Figure 3. Subsequently, we computed module eigengenes, which indicate the total gene expression level of each module and were grouped based on their association. Three modules were identified, as shown in Figure 5B. Only one module was correlated with SCZ (turquoise; cor = -0.27, *P* = 0.01). The 64 genes related with SCZ identified in this module were maintained for future investigation, as shown in Figures 5C,D. Finally, eight genes were determined to overlap between DEGs and the selected Genes in MEturquoise and are also shown in Figure 5E.

### Hub gene identification

**FIGURE 6:**
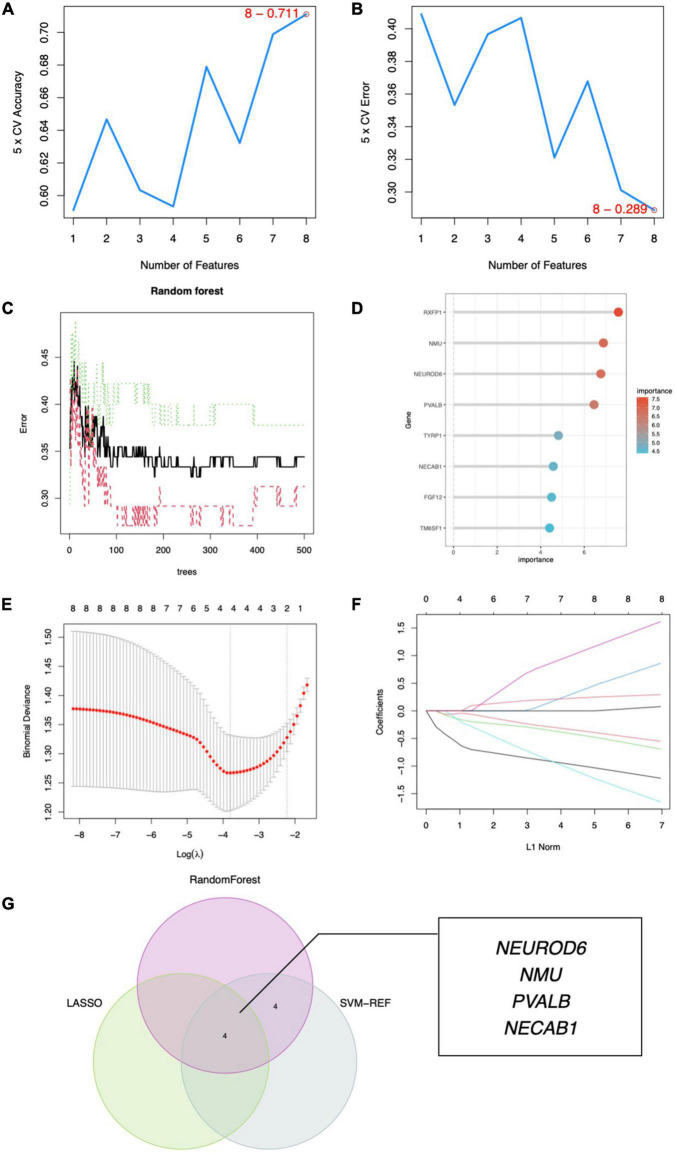
Hub gene identification. **(A)** Eight gene signatures were identified by SVM-RFE analysis with an accuracy of 0.711. **(B)** Error of 0.289. **(C)** Prediction accuracy of the RF model. **(D)** Gene importance scores of RF model. **(E)** Cross-validation to select the optimal tuning parameter log (Lambda) in LASSO regression analysis. **(F)** LASSO coefficient profiles of candidate genes. **(G)** Venn diagram of four hub genes shared by the SVM-RFE, RF, and LASSO algorithms.

**FIGURE 7:**
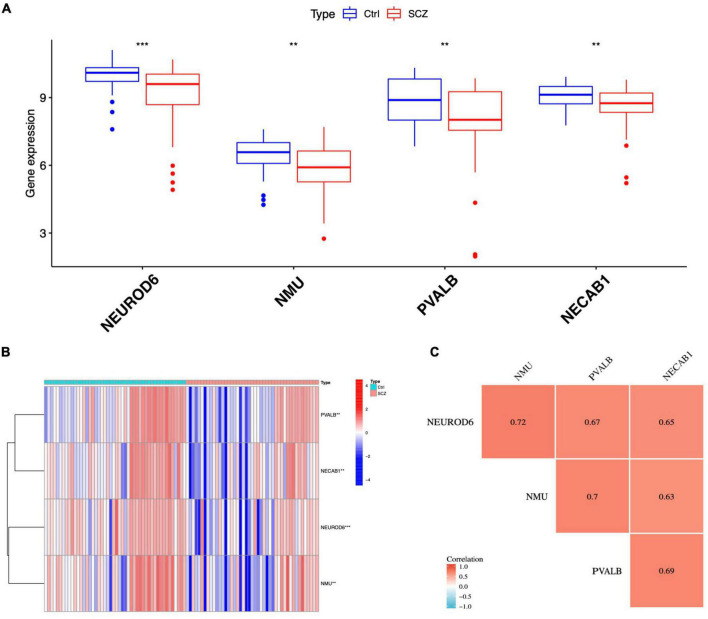
Expression analysis of hub genes. **(A)** Expression of four hub genes in SCZ and control groups. **(B)** Heatmap of hub gene expression. **(C)** Correlation between hub genes. **P < 0.01, ***P < 0.001 vs. Ctrl.

To discover gene signatures, the eight candidate genes were submitted into SVM-RFE, RF, and LASSO. We identified an eight-gene signature using SVM with a precision of 0.711, as shown in Figures 6A,B. The random forest method sorted eight genes with importance scores greater than four, as shown in Figures 6C,D. LASSO regression analyses identified four gene signatures, as shown in Figures 6E,F. To obtain a robust gene signature for SCZ, we determined which genes overlapped from the three methods and obtained four hub genes: NEUROD6, NMU, PVALB, and NECAB1, as shown in Figure 6G. NEUROD6, NMU, PVALB, and NECAB1 were significantly decreased in SC samples compared to control, as shown in Figures 7A,B. Correlation analysis showed that the four genes had robust positive correlations with each other, as shown in Figure 7C.

### Gene set enrichment of the hub genes

**FIGURE 8:**
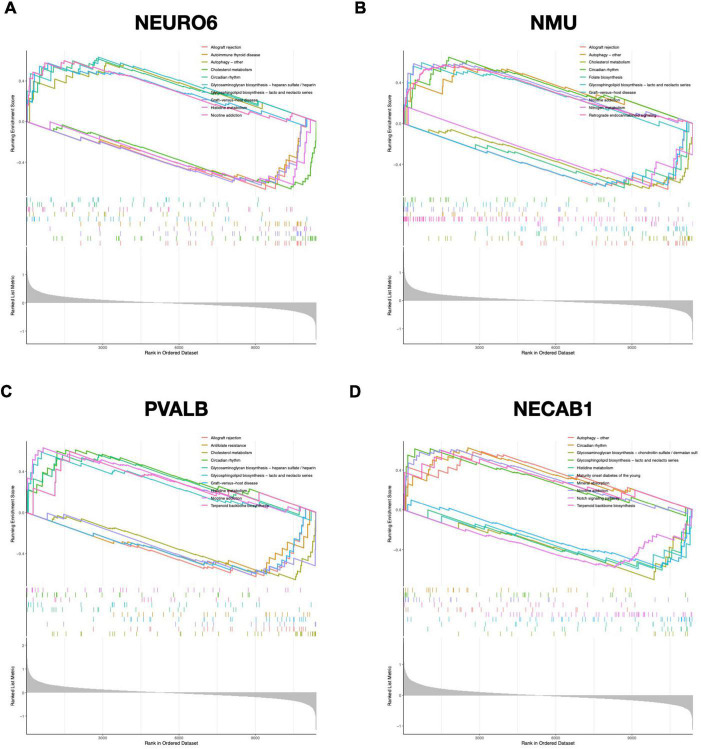
GSEA analysis of hub genes. Top 5 GSEA enrichment in the high and low expression gene set of **(A)** NEURO6, **(B)** NMU, **(C)** PVALB, and **(D)** NECAB1.

To further uncover the probable roles of NEUROD6, NMU, PVALB, and NECAB1, we conducted GSEA. Genes in the low expression categories of the four hub genes were significantly enriched in allograft rejection, autoimmune thyroid disease, graft vs. host disease, antifolate resistance, and glycosaminoglycan biosynthesis, as shown in Figure 8.

### Correlation of hub genes and immunocyte infiltration

**FIGURE 9:**
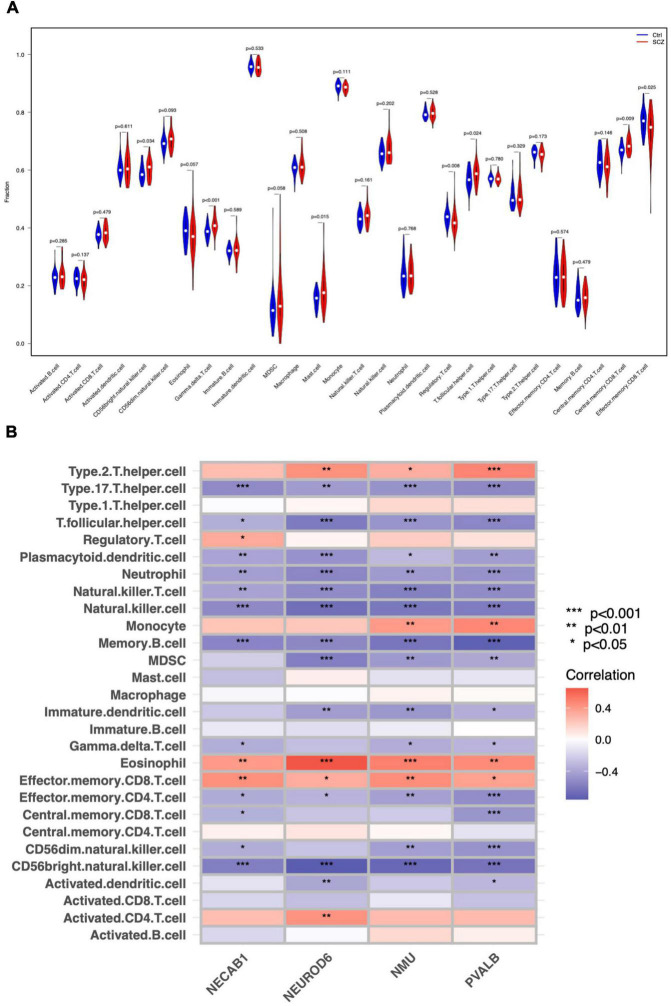
Immune cell distribution in SCZ. **(A)** Differences in infiltrated immune cells between SCZ and control groups. **(B)** Correlation analysis between hub genes and immune cells.

We investigated the pattern of immunocytes infiltration using ssGSEA and found that the abundance of CD56 bright NK cells, gamma delta T cells, mast cells, follicular T helper cells, and central memory CD8 + T cells were much greater in SCZ samples than in normal samples, whereas the regulatory T cells and effector memory CD8 + T cells was significantly reduced, as shown in Figure 9A and Supplementary Figure 4. Furthermore, we calculated the correlation between hub gene expression and infiltrating immune cells, and the results showed that most immunocytes had a significant negative connection with hub genes, as shown in Figure 9B. These results imply that the inflammatory components may play an essential role in the development of SCZ, and hub genes may have a novel regulatory role in immune function.

### Diagnostic model construction

**FIGURE 10:**
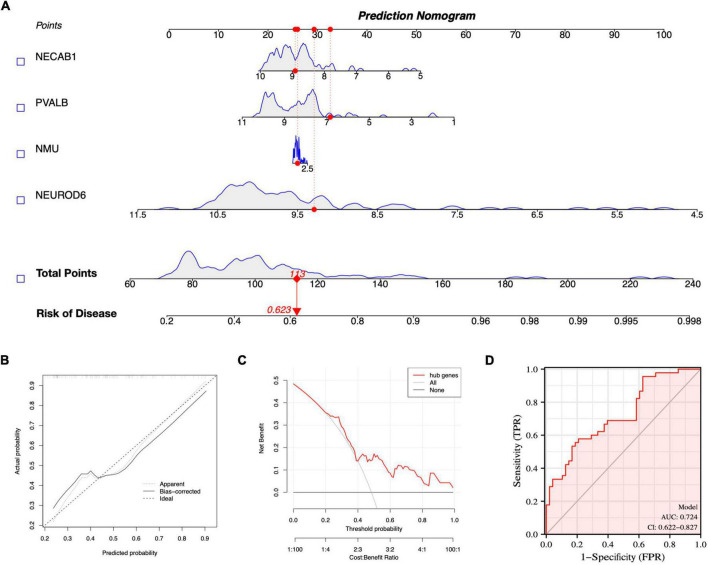
Nomogram model construction for SCZ diagnosis. **(A)** Nomogram to predict SCZ risk. **(B)** Calibration curve evaluation for the diagnostic potential of the nomogram model. **(C)** DCA curve to assess the nomogram practical efficacy. **(D)** ROC curve to evaluate prediction accuracy.

A nomogram model for SCZ diagnosis was established based on NEUROD6, NMU, PVALB, and NECAB1, as shown in Figure 10A. The calibration curve indicated that the variance between observed and predicted risk was limited, indicating that the nomogram model performed very well in predicting SCZ, as shown in Figure 10B. At the risk threshold of 0.1–1.0, DCA showed that the hub genes curve was above the gray line and indicated a significant net benefit from using nomograms to forecast SCZ risk, as shown in Figure 10C. The AUC of the nomogram reached at 0.724, and the 95% confidence interval (CI) ranged from 0.622 to 0.827, as shown in Figure 10D. High-accuracy risk prediction of the diagnostic nomogram for SCZ was observed.

### Diagnostic evaluation of hub genes

**FIGURE 11:**
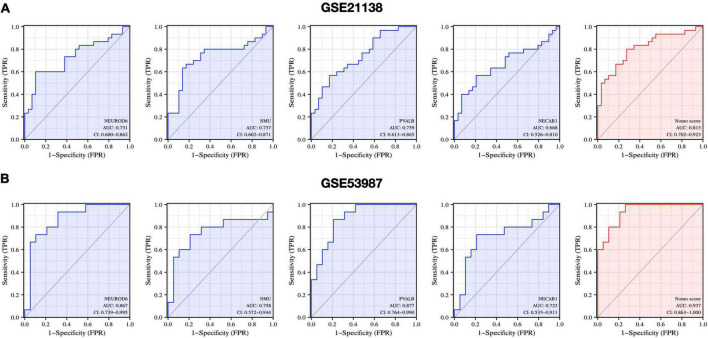
Diagnostic evaluation of hub genes and nomogram score. ROC curve to evaluate prediction accuracy in **(A)** GSE21138 and **(B)** GSE53987.

We further evaluated the diagnostic values of the four hub genes (NEUROD6, NMU, PVALB, and NECAB1) and nomogram model scores in GSE21138 and GSE53987 using ROC curves. The AUC values of hub genes in SCZ and healthy samples measured in GSE21138 were NEUROD6:0.731 (95% CI, 0.600–0.862), NMU: 0.737 (95% CI, 0.602–0.871), PVALB: 0.739 (95% CI, 0.613–0.865), and NECAB1:0.668 (95% CI, 0.526–0.810). The AUC of the nomogram model score was 0.813 (95% CI, 0.702–0.923), as shown in Figure 11A. In GSE53987, the AUC of hub genes were NEUROD6: 0.867 (95% CI, 0.739–0.995), NMU: 0.758 (95% CI, 0.572–0.994), PVALB: 0.877 (95% CI, 0.764–0.990), and NECAB1: 0.723 (95% CI, 0.535–0.911). The AUC of the nomogram model score was 0.937 (95% CI, 0.863–1.000), as shown in Figure 11B. These results indicate that the four hub genes may have significant diagnostic value for SCZ. The AUC of the nomogram model based on the four hub genes was larger than that of a single hub gene, suggesting that when hub genes were regarded together, the diagnostic value was greater, which is more conducive to clinical SCZ prediction.

### Hub gene validation

**FIGURE 12:**
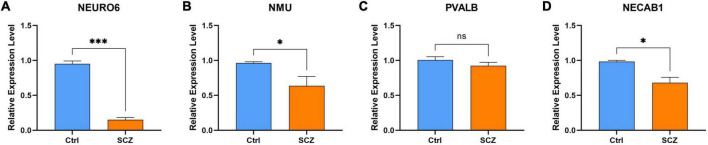
qPCR validation. Comparison of gene expression between SCZ rat model and control in **(A)** NEURO6, **(B)** NMU, **(C)** PVALB, and **(D)** NECAB1. *P < 0.05, ^***^P < 0.001 vs. Ctrl.

The experimental design was approved by the Animal Ethics Committee of the First Hospital of Jilin University (NO: 20210637). NEURO6, NMU, and NECAB1 were downregulated in the MK801-induced SCZ rat model compared to the control. Nevertheless, no significant changes were observed in PVALB expression, as shown in Figure 12.

### Drug prediction for schizophrenia treatment

**FIGURE 13:**
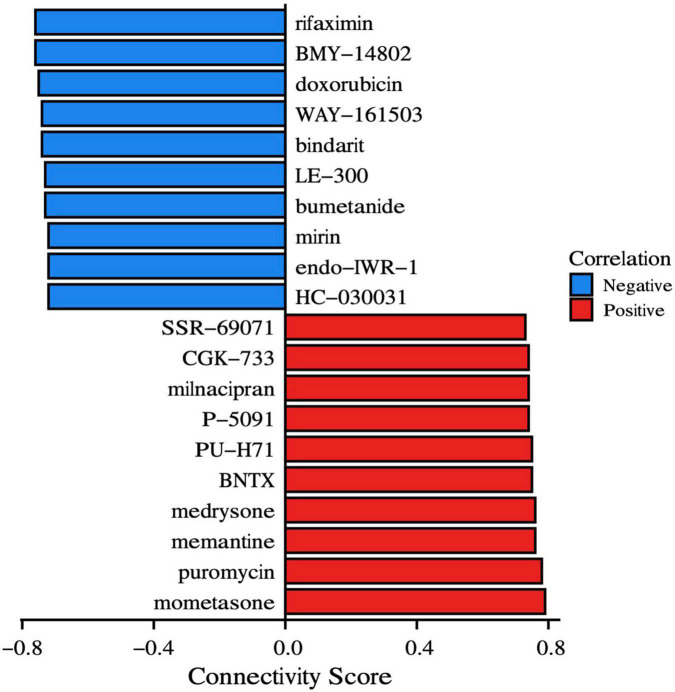
Drugs prediction. CMap instances organized by compounds and cell lines depict the most significant positive and negative correlations to the impact on SCZ. The connection score, shown on the x-axis, represents the strength of the association.

Differentially expressed mRNAs in SCZ were submitted to the CMap database to predict prospective small-molecule compounds with SCZ therapeutic potential. Drugs exhibiting negative correlations have the potential to improve SCZ symptoms. The top ten small molecular drugs (all connectivity scores > 0.7) are shown in Figure 13. Among these, LE-300 is a dopamine receptor antagonist, WAY-161503 is a serotonin receptor agonist, and endo-IWR-1 is an inhibitor of the Wnt/-catenin signaling pathway.

## Discussion

SCZ is a psychiatric disorder with high global prevalence and a multifactorial biological etiology. The mechanics of SCZ are yet to be completely understood. Clinical manifestations of SCZ include hallucinations, disordered emotions, and social isolation caused by disruptions of the immunological, metabolic, and endocrine systems (McCutcheon et al., 2020). Owing to the likelihood of developing disability and rising prevalence, SCZ not only significantly impacts patient health but also has major public health implications (Carpenter and Buchanan, 1994). Therefore, understanding the critical pathways and gene signatures in SCZ could aid in the assessment of risk evaluation, pathogenesis, and personalized therapy.

In this work, we conducted a comprehensive examination of SCZ using PFC samples, which was revealed as a hub region correlated with SCZ. Impaired cognitive functions and aberrant behaviors have been consistently implicated in patients with SCZ with decreased PFC volumes (Tomasik et al., 2016). Despite the complexity of PFC functional regions, the connectivity between the basal ganglia, inferior frontal gyrus, hippocampus, lateral habenular, and other brain atlases is characterized by extremely complex anatomical networks and variability in behavior and activation (Zhou et al., 2015; Brady et al., 2019; Mathis et al., 2021). However, both hypo- and hyperfrontality have been hypothesized as valid and informative reflections of PFC dysfunction in SCZ. The basis of this dysfunction and its exact contributions remain unclear (Manoach, 2003). These variables may also impact the co-prevalence of certain autoimmune illnesses and some instances of SCZ (Tomasik et al., 2016).

Cells of the immune system and the central nervous system can interact. The immune system responds to infection, tissue damage and trauma by releasing substances that trigger an inflammatory response. Inflammatory cytokines released by the immune system are considered to be a key feature of neurological pathology, such as chronic pain, neurodegenerative diseases, spinal cord injuries, and neuropsychiatric disorders, particularly SCZ (Skaper et al., 2014). Immune infiltration impairment was found in the SCZ PFC, which is consistent with previous findings (Maas et al., 2017). A higher CD56 bright NK cells proportion has been observed in SCZ patients, with activation to secrete TNF-α and IFN-γ, which causes damage to the central nervous system (Miller et al., 2013). Mast cell infiltration may also affect cognitive performance (Skaper et al., 2014). Increased gamma/delta T lymphocytes in unmedicated patients with SCZ impair the blood–brain barrier (Wo et al., 2020). Abundant increases in any of these cell types of influence SCZ pathology. Moreover, the number of regulatory T cells (Treg) was found to decrease in SCZ samples. Inflammatory disorders and SCZ have been linked throughout the recent decades. Immunological-mediated neuropathology is a rising issue, and new research emphasizes the significance of innate immune signaling in SCZ (Hartwig et al., 2017; Yang and Tsai, 2017). Tregs may contribute to the improvement of negative symptoms in SCZ (Kelly et al., 2018). Elevated Tregs in SCZ are correlated with fewer negative symptoms, possibly by counteracting ongoing inflammatory processes (Corsi-Zuelli and Deakin, 2021). Recent studies have demonstrated regulatory connections between microglia, astrocytes, and Tregs. Treg cell dysfunction relates to glial damage, low-level inflammation, and reduced life expectancy in SCZ (Kelly et al., 2018; Corsi-Zuelli et al., 2021). Tregs are also capable of promoting oligodendrocyte differentiation and (re)myelination. Treg knockout mice had markedly reduced remyelination and oligodendrocyte differentiation, resulting in cognitive impairment (Dombrowski et al., 2017). Our study revealed the immune infiltration landscape of SCZ, which paved the way for designing immunotherapy for SCZ based on molecular alterations.

For decades, pathophysiological investigations on SCZ have concentrated on dopaminergic and glutamatergic neurotransmission abnormalities, with scant clinical advancements. Microarray data may now be utilized to uncover hub genes, interaction networks, and pathways that interpret SCZ, according to the tremendous progress of bioinformatics. In this study, DEGs were mainly enriched for neurofunctional activities. The CMap database predicted highly correlated molecular drugs (connectivity scores > 0.7) for SCZ treatment. Bumetanide is a selective antagonist of Na-K-Cl cotransporter (NKCC1) which can reduce intracellular chloride concentrations and enhances the inhibitory effect of GABAergic neurons, an FDA-approved drug with the potential to treat or prevent cognitive impairment in SCZ syndrome (Lemonnier et al., 2016). CCL2 levels are significantly higher in patients with SCZ, dysregulated CCL2 may be one of the important reasons for the negative symptoms of SCZ, and the duration of the disease is closely related to the negative symptoms (Hong et al., 2017). CCL2 inhibition by oral administration of bindarit may potentially improve symptoms of SCZ (Raghu et al., 2017). Rifaximin can reduce gut-derived inflammation, which may also contribute to the relief of SCZ (Li H. et al., 2021; Patel et al., 2022). The brain-gut axis refers to the two-way communication network between the brain and the gut. Different signals from the gastrointestinal tract can regulate brain function through neural, endocrine, immune, metabolic and other pathways (Lach et al., 2018). When the types of intestinal microbes change, the central nervous system will also change accordingly, which is mainly due to the change of metabolites of intestinal microbes (Erny et al., 2015). Although the reasons for this are not fully understood, altering the gut microbiome can improve mental activity, emotional and cognitive processes and behavior in animals (Zheng et al., 2016; Valles-Colomer et al., 2019). Further analyses are necessary to analyze the effect of these molecules on behavioral tests in animal models of SCZ and patients with SCZ.

Based on bioinformatics methods, this is the first study to find four hub genes closely related to SCZ (NEUROD6, NMU, PVALB, and NECAB1). Enrichment and immune infiltration analyses showed that these genes might lead to the onset of SCZ by regulating the genetic process of cells or affecting the immune environment. The NEUROD family (NEUROD1, 2, 4, 6) is a key regulator of neural progenitor cell differentiation (Tutukova et al., 2021). NEUROD6 plays a role in cytoskeletal protein function, mitochondrial trafficking, membrane potential regulation, and mitochondrial chaperoning (Cherry et al., 2011). NEUROD6 may also improve cellular resistance to oxidative stress, which is important in neurodegenerative illness prevention including Parkinson’s disease and autism spectrum disorder (Viereckel et al., 2016). Recently, NEUROD6 was revealed as a potential biomarker for of Alzheimer’s disease (AD) diagnosis. Alzheimer’s animal models and postmortem Alzheimer’s patients have both shown low levels of NEUROD6 expression (Satoh et al., 2014). Locomotion was dramatically increased in NEUROD6-KO mice with repeated psychostimulant administration, and optogenetic stimulation of NEUROD6-Cre VTA neurons was found to trigger glutamatergic postsynaptic currents as well as DA secretion in nucleus accumbens (Bimpisidis et al., 2019). Information processing is affected by γ-aminobutyric acid (GABA) neurons that produce parvalbumin, somatostatin, or vasoactive intestinal peptides (Fachim et al., 2018). Parvalbumin (PVALB) deficits or downregulation are common in patients with SCZ (Tsubomoto et al., 2019). PV-deficient (PV-/-) mice exhibit a strong autism-like behavioral phenotype. PVALB neurons exhibit precise control over spike timing, leading to the formation and regulation of gamma rhythms, which are necessary for sensory perception and awareness (Janickova et al., 2021). NMDA receptor hypofunction in pyramidal cells results in decreased activity of PVALB neurons, thus reducing network gamma oscillatory activity (Kaur et al., 2020). Neuromedin U (NMU) is widely distributed neuropeptide. NMU is involved in physiological activities, including feeding behavior, metabolism, physiological stress, circadian rhythmicity, and inflammatory processes (Ly and Root, 2021; Sasaki-Hamada et al., 2021), as well as reward circuits (Anan et al., 2020). The ability of neuropeptides to reduce food intake in rodents prompted the modification of peptide ligands (Vallöf et al., 2020). NMU receptor activation promotes GABAergic neurons in the hippocampus (Ghashghayi et al., 2022). Recent research indicates that NMU may regulate psychomotor activity. NMU-21 elicits anxiolytic-like effects in the goldfish brain (Matsuda et al., 2020). As the major calcium-binding protein in CB1/CCK-positive interneurons, neuronal calcium-binding protein 1 (NECAB1) is found in several excitatory neuron populations in the rat spinal cord. The soma volume of pyramidal cells immunoreactive for NECAB1 is significantly reduced in SCZ (Maldonado-Avilés et al., 2006).

Correlation analysis found that the four hub genes were highly positively correlated with one another (all cor > 0.6). Therefore, we suspect that they play a vital role in SCZ pathology. Furthermore, we established a nomogram model for SCZ risk prediction. The diagnosis and treatment of SCZ currently are mostly dependent on clinical surveys with inadequate response rates. Recurrence of symptoms is typical among people who cease treatment. Precise diagnosis and early precautions for SCZ are essential to help reduce suffering and enhance the prognosis of the condition. In the present two datasets of SCZ, the AUCs of the four hub genes were greater than 0.65. These results suggest that NEUROD6, NMU, PVALB, and NECAB1 have good diagnostic values. Moreover, the AUC of the nomogram model reached 0.813 (GSE21138) and 0.937 (GSE53987), which exhibited excellent accuracy for disease prediction. Collectively, NEUROD6, NMU, PVALB, NECAB1, and the nomogram model of the four hub genes have great potential as diagnostic biomarkers and therapeutic targets for SCZ.

This study was subjected to the following limitations. First, four hub genes were identified by qPCR, and their localization and distribution should be verified, and the model method may cannot completely simulate SCZ. This may be one of the reasons that the results of qPCR were not completely consistent with expectations. Second, the scope of this study was insufficient to include detailed validation of *in vivo* and *in vitro*. Third, more clinical and demographic characteristics of patients with SCZ should be included in the study for further subgroup analysis.

## Conclusion

Based on bioinformatics methods, a gene signature of NEUROD6, NMU, PVALB, and NECAB1 that intimately associated to SCZ were initially identified. A predictive nomogram for the clinical diagnosis of SCZ was established. This predictive nomogram can be applied clinically to identify patients at high risk of SCZ.

## Data availability statement

The original contributions presented in this study are included in the article/Supplementary material, further inquiries can be directed to the corresponding author/s.

## Ethics statement

The animal study was reviewed and approved by the Animal Ethics Committee of The First Hospital of Jilin University (Approval No. 20210637).

## Author contributions

HM, MC, and CZ: conceptualization. SX: methodology. MC: software. ND: validation. CZ and HM: writing—original draft preparation. All authors have read and agreed to the published version of the manuscript.

## Footnotes

1
https://www.ncbi.nlm.nih.gov/geo/query/acc.cgi?acc=gse21138

2
https://www.ncbi.nlm.nih.gov/geo/query/acc.cgi?acc=gse53987

3
https://clue.io/
