# Supplementary material for: Identification of hub genes and construction of diagnostic nomogram model in schizophrenia
Source: Front Aging Neurosci. 2022 Oct 14;14:1032917. doi: 10.3389/fnagi.2022.1032917 (PMC9614240; doi:10.3389/fnagi.2022.1032917)
Supplement: Supplementary file 1 [file Data_Sheet_1.PDF]

Supplementary Table 1. GEO datasets of schizophrenia for analysis.

GEO ID	Platform	Tissue types	Patients	Control
GSE21138	GPL570	Prefrontal cortex	30	29
GSE53987	GPL570	Prefrontal cortex	15	19
Total			45	48
