# Supplementary material for: Identification of hub genes and construction of diagnostic nomogram model in schizophrenia
Source: Front Aging Neurosci. 2022 Oct 14;14:1032917. doi: 10.3389/fnagi.2022.1032917 (PMC9614240; doi:10.3389/fnagi.2022.1032917)
Supplement: Supplementary file 2 [file Data_Sheet_2.PDF]

Supplementary Table 2. GO enrichment

ONTOLOGY	ID	Description	BgRatio	pvalue	p.adjust	qvalue
BP	GO:0098840	sport along	12/18800	9.38E-05	0.03737621	0.02789085
BP	GO:0099118	-based prote	12/18800	9.38E-05	0.03737621	0.02789085
BP	GO:0007218	tide signalin	108/18800	0.00030029	0.07285658	0.05436699
BP	GO:0097193	ptotic signal	295/18800	0.00041565	0.07285658	0.05436699
BP	GO:0060259	n of feeding	26/18800	0.00045707	0.07285658	0.05436699
BP	GO:0030534	dult behavio	136/18800	0.00058981	0.07834692	0.05846399
BP	GO:0007568	aging	163/18800	0.00099752	0.08298926	0.06192819
BP	GO:0006882	zinc ion hom	39/18800	0.00103209	0.08298926	0.06192819
BP	GO:0021543	um developr	169/18800	0.00110721	0.08298926	0.06192819
BP	GO:0055069	ion homeos	41/18800	0.00114042	0.08298926	0.06192819
BP	GO:2001242	sic apoptotic	171/18800	0.0011454	0.08298926	0.06192819
BP	GO:0009266	o temperatu	180/18800	0.00132757	0.08817253	0.06579605
BP	GO:0010043	onse to zinc	53/18800	0.00189945	0.10966641	0.0818352
BP	GO:0044703	ism reprodu	205/18800	0.00192639	0.10966641	0.0818352
BP	GO:0044706	ellular orgar	213/18800	0.0021483	0.11414627	0.08517815
BP	GO:0050795	ation of beh	69/18800	0.00319555	0.15183226	0.11330017
BP	GO:0021537	halon devel	247/18800	0.00326694	0.15183226	0.11330017
BP	GO:0021766	ampus devel	80/18800	0.00426933	0.15183226	0.11330017
BP	GO:0006446	of translation	81/18800	0.00437416	0.15183226	0.11330017
BP	GO:2001243	ntrinsic apop	102/18800	0.00684668	0.15183226	0.11330017
BP	GO:0007631	eding behavi	106/18800	0.00737507	0.15183226	0.11330017
BP	GO:0021761	ystem devel	108/18800	0.00764602	0.15183226	0.11330017
BP	GO:0009408	sponse to he	109/18800	0.00778317	0.15183226	0.11330017
BP	GO:0001933	on of protein	341/18800	0.00800407	0.15183226	0.11330017
BP	GO:0002526	flammatory r	113/18800	0.00834288	0.15183226	0.11330017
BP	GO:0018958	g compound	113/18800	0.00834288	0.15183226	0.11330017
BP	GO:0046916	ion metal ior	115/18800	0.00862937	0.15183226	0.11330017
BP	GO:0010038	onse to meta	351/18800	0.00866219	0.15183226	0.11330017
BP	GO:0045471	onse to etha	119/18800	0.0092155	0.15183226	0.11330017
BP	GO:0006413	lational initi	120/18800	0.00936476	0.15183226	0.11330017
BP	GO:2001233	poptotic sigr	370/18800	0.0099988	0.15183226	0.11330017
BP	GO:0030900	rain develop	376/18800	0.01044464	0.15183226	0.11330017
BP	GO:0042326	lation of pho	382/18800	0.01090198	0.15183226	0.11330017
BP	GO:0001701	nbryonic dev	387/18800	0.01129193	0.15183226	0.11330017
BP	GO:0008343	feeding beh	10/18800	0.01216981	0.15183226	0.11330017
BP	GO:0048563	animal orgar	10/18800	0.01216981	0.15183226	0.11330017
BP	GO:1902224	ody metaboli	10/18800	0.01216981	0.15183226	0.11330017
BP	GO:1905383	alization to p	10/18800	0.01216981	0.15183226	0.11330017
BP	GO:0006986	e to unfoldec	139/18800	0.01240389	0.15183226	0.11330017
BP	GO:0051384	se to glucoco	139/18800	0.01240389	0.15183226	0.11330017
BP	GO:0055076	metal ion ho	139/18800	0.01240389	0.15183226	0.11330017
BP	GO:0097084	smooth mus	11/18800	0.01337897	0.15183226	0.11330017
BP	GO:0106049	ular response	11/18800	0.01337897	0.15183226	0.11330017
BP	GO:2000253	ation of feec	11/18800	0.01337897	0.15183226	0.11330017

BP	GO:0002674	of acute infl	12/18800	0.01458671	0.15183226	0.11330017
BP	GO:0035865	ɔonse to pot	12/18800	0.01458671	0.15183226	0.11330017
BP	GO:0097201	m RNA polyı	12/18800	0.01458671	0.15183226	0.11330017
BP	GO:2001214	ılation of va:	12/18800	0.01458671	0.15183226	0.11330017
BP	GO:0007189	ɔrotein-coup	155/18800	0.01525389	0.15183226	0.11330017
BP	GO:0010970	rt along micr	157/18800	0.01562827	0.15183226	0.11330017
BP	GO:0031960	se to cortico	157/18800	0.01562827	0.15183226	0.11330017
BP	GO:0015816	ʏcine transpo	13/18800	0.01579304	0.15183226	0.11330017
BP	GO:0045820	lation of glyc	13/18800	0.01579304	0.15183226	0.11330017
BP	GO:0060670	labyrinthine	13/18800	0.01579304	0.15183226	0.11330017
BP	GO:0070486	ɔcyte aggreg	13/18800	0.01579304	0.15183226	0.11330017
BP	GO:0071474	yperosmotic	13/18800	0.01579304	0.15183226	0.11330017
BP	GO:1901862	ı of muscle t	13/18800	0.01579304	0.15183226	0.11330017
BP	GO:0045936	of phosphat	440/18800	0.01592696	0.15183226	0.11330017
BP	GO:0010563	ɔf phosphorı	441/18800	0.01602334	0.15183226	0.11330017
BP	GO:0032103	of response	442/18800	0.01612004	0.15183226	0.11330017
BP	GO:0035966	ɔologically ir	160/18800	0.01619726	0.15183226	0.11330017
BP	GO:0002864	matory respo	14/18800	0.01699795	0.15183226	0.11330017
BP	GO:0009886	ıic animal m	14/18800	0.01699795	0.15183226	0.11330017
BP	GO:0032352	of hormone	14/18800	0.01699795	0.15183226	0.11330017
BP	GO:0042117	ıocyte activa	14/18800	0.01699795	0.15183226	0.11330017
BP	GO:0043455	econdary me	14/18800	0.01699795	0.15183226	0.11330017
BP	GO:0046459	ıtty acid meta	14/18800	0.01699795	0.15183226	0.11330017
BP	GO:0047484	response to	14/18800	0.01699795	0.15183226	0.11330017
BP	GO:0048021	ıelanin biosy	14/18800	0.01699795	0.15183226	0.11330017
BP	GO:1900376	ry metabolite	14/18800	0.01699795	0.15183226	0.11330017
BP	GO:0023061	;ignal release	451/18800	0.01700544	0.15183226	0.11330017
BP	GO:0071466	nse to xenob	168/18800	0.01775762	0.15183226	0.11330017
BP	GO:0002031	ɔled receptor	15/18800	0.01820146	0.15183226	0.11330017
BP	GO:0007567	parturition	15/18800	0.01820146	0.15183226	0.11330017
BP	GO:0010273	cation of cop	15/18800	0.01820146	0.15183226	0.11330017
BP	GO:1990169	;ponse to co	15/18800	0.01820146	0.15183226	0.11330017
BP	GO:2000767	on of cytopla	15/18800	0.01820146	0.15183226	0.11330017
BP	GO:2001028	ı of endothe	15/18800	0.01820146	0.15183226	0.11330017
BP	GO:0006417	ıtion of trans	463/18800	0.0182282	0.15183226	0.11330017
BP	GO:0017014	ein nitrosyla	16/18800	0.01940355	0.15183226	0.11330017
BP	GO:0018119	ysteine S-nit	16/18800	0.01940355	0.15183226	0.11330017
BP	GO:0032095	n of respons	16/18800	0.01940355	0.15183226	0.11330017
BP	GO:0035864	se to potassi	16/18800	0.01940355	0.15183226	0.11330017
BP	GO:0035970	onine depho	16/18800	0.01940355	0.15183226	0.11330017
BP	GO:0042448	one metabol	16/18800	0.01940355	0.15183226	0.11330017
BP	GO:0051238	;tering of me	16/18800	0.01940355	0.15183226	0.11330017
BP	GO:0061684	ɔ-mediated a	16/18800	0.01940355	0.15183226	0.11330017
BP	GO:0071391	ɔnse to estro	16/18800	0.01940355	0.15183226	0.11330017
BP	GO:2001212	ɔn of vasculo	16/18800	0.01940355	0.15183226	0.11330017
BP	GO:0002791	n of peptide	180/18800	0.02021338	0.15183226	0.11330017

BP	GO:2001257	f cation cha	180/18800	0.02021338	0.15183226	0.11330017
BP	GO:0002544	flammatory	17/18800	0.02060424	0.15183226	0.11330017
BP	GO:0010566	etone biosy	17/18800	0.02060424	0.15183226	0.11330017
BP	GO:0015812	inobutyric a	17/18800	0.02060424	0.15183226	0.11330017
BP	GO:0021542	gyrus devel	17/18800	0.02060424	0.15183226	0.11330017
BP	GO:0046827	of protein e	17/18800	0.02060424	0.15183226	0.11330017
BP	GO:0048569	animal orga	17/18800	0.02060424	0.15183226	0.11330017
BP	GO:0051969	ansmission c	17/18800	0.02060424	0.15183226	0.11330017
BP	GO:0008217	on of blood	183/18800	0.0208485	0.15183226	0.11330017
BP	GO:0045766	ulation of a	183/18800	0.0208485	0.15183226	0.11330017
BP	GO:0090087	of peptide	183/18800	0.0208485	0.15183226	0.11330017
BP	GO:1904018	n of vasculat	183/18800	0.0208485	0.15183226	0.11330017
BP	GO:0007565	nale pregna	185/18800	0.02127656	0.15183226	0.11330017
BP	GO:0072503	inorganic cat	494/18800	0.02161161	0.15183226	0.11330017
BP	GO:0007625	oming beha	18/18800	0.02180352	0.15183226	0.11330017
BP	GO:0043116	tion of vasc	18/18800	0.02180352	0.15183226	0.11330017
BP	GO:0061687	of inorgani	18/18800	0.02180352	0.15183226	0.11330017
BP	GO:0009755	diated signa	190/18800	0.0223628	0.15183226	0.11330017
BP	GO:0071248	esponse to r	191/18800	0.02258279	0.15183226	0.11330017
BP	GO:0048839	ear develop	192/18800	0.02280369	0.15183226	0.11330017
BP	GO:0002523	volved in in	19/18800	0.0230014	0.15183226	0.11330017
BP	GO:0010801	eptidyl-thre	19/18800	0.0230014	0.15183226	0.11330017
BP	GO:0045947	ion of transl	19/18800	0.0230014	0.15183226	0.11330017
BP	GO:0060546	ation of necr	19/18800	0.0230014	0.15183226	0.11330017
BP	GO:0097501	sponse to m	19/18800	0.0230014	0.15183226	0.11330017
BP	GO:0030705	endent intra	197/18800	0.02392172	0.15183226	0.11330017
BP	GO:0099111	ule-based t	198/18800	0.02414801	0.15183226	0.11330017
BP	GO:0006700	rmone biosy	20/18800	0.02419787	0.15183226	0.11330017
BP	GO:0042053	opamine me	20/18800	0.02419787	0.15183226	0.11330017
BP	GO:0042069	echolamine r	20/18800	0.02419787	0.15183226	0.11330017
BP	GO:0062099	f programm	20/18800	0.02419787	0.15183226	0.11330017
BP	GO:1902176	ss-induced i	20/18800	0.02419787	0.15183226	0.11330017
BP	GO:0002029	n-coupled r	21/18800	0.02539295	0.15183226	0.11330017
BP	GO:0022401	ation of sign	21/18800	0.02539295	0.15183226	0.11330017
BP	GO:0032098	lation of app	21/18800	0.02539295	0.15183226	0.11330017
BP	GO:0035809	ion of urine	21/18800	0.02539295	0.15183226	0.11330017
BP	GO:0046716	ll cellular ho	21/18800	0.02539295	0.15183226	0.11330017
BP	GO:1900543	urine nucle	21/18800	0.02539295	0.15183226	0.11330017
BP	GO:0023058	of signalin	22/18800	0.02658662	0.15183226	0.11330017
BP	GO:0045980	of nucleotid	22/18800	0.02658662	0.15183226	0.11330017
BP	GO:0046885	rmone biosy	22/18800	0.02658662	0.15183226	0.11330017
BP	GO:0060713	e layer morp	22/18800	0.02658662	0.15183226	0.11330017
BP	GO:2000310	f NMDA rece	22/18800	0.02658662	0.15183226	0.11330017
BP	GO:0006457	rotein foldin	212/18800	0.02740879	0.15183226	0.11330017
BP	GO:0006469	ion of protei	213/18800	0.02764823	0.15183226	0.11330017
BP	GO:0010893	of steroid bi	23/18800	0.02777889	0.15183226	0.11330017

BP	GO:0032331	ı of chondro	23/18800	0.02777889	0.15183226	0.11330017
BP	GO:0042438	biosynthetic	23/18800	0.02777889	0.15183226	0.11330017
BP	GO:0060575	thelial cell di	23/18800	0.02777889	0.15183226	0.11330017
BP	GO:0006582	ı metabolic	24/18800	0.02896977	0.15183226	0.11330017
BP	GO:0042026	otein refoldi	24/18800	0.02896977	0.15183226	0.11330017
BP	GO:0048011	K receptor si	24/18800	0.02896977	0.15183226	0.11330017
BP	GO:0060547	ation of nec	24/18800	0.02896977	0.15183226	0.11330017
BP	GO:0070841	on body ass	24/18800	0.02896977	0.15183226	0.11330017
BP	GO:0070935	iated mRNA	24/18800	0.02896977	0.15183226	0.11330017
BP	GO:0071294	response to	24/18800	0.02896977	0.15183226	0.11330017
BP	GO:0120255	ound biosyn	24/18800	0.02896977	0.15183226	0.11330017
BP	GO:2001026	endothelial c	24/18800	0.02896977	0.15183226	0.11330017
BP	GO:0043583	r developme	219/18800	0.02910278	0.15183226	0.11330017
BP	GO:0071241	ıse to inorga	221/18800	0.0295944	0.15183226	0.11330017
BP	GO:0002438	response tc	25/18800	0.03015925	0.15183226	0.11330017
BP	GO:0032104	onse to extr	25/18800	0.03015925	0.15183226	0.11330017
BP	GO:0032107	response to	25/18800	0.03015925	0.15183226	0.11330017
BP	GO:0044550	abolite biosy	25/18800	0.03015925	0.15183226	0.11330017
BP	GO:0048520	egulation of	25/18800	0.03015925	0.15183226	0.11330017
BP	GO:0002790	ptide secreti	225/18800	0.03058771	0.15183226	0.11330017
BP	GO:0002026	ıe force of he	26/18800	0.03134734	0.15183226	0.11330017
BP	GO:0006972	osmotic resp	26/18800	0.03134734	0.15183226	0.11330017
BP	GO:0030318	ocyte differer	26/18800	0.03134734	0.15183226	0.11330017
BP	GO:0048641	etal muscle ti	26/18800	0.03134734	0.15183226	0.11330017
BP	GO:0071549	e to dexamet	26/18800	0.03134734	0.15183226	0.11330017
BP	GO:0071356	se to tumor	229/18800	0.03159431	0.15183226	0.11330017
BP	GO:2001234	of apoptoti	230/18800	0.03184803	0.15183226	0.11330017
BP	GO:0001963	ısmission, do	27/18800	0.03253403	0.15183226	0.11330017
BP	GO:0010460	egulation of	27/18800	0.03253403	0.15183226	0.11330017
BP	GO:0060669	placenta mor	27/18800	0.03253403	0.15183226	0.11330017
BP	GO:0071280	esponse to co	27/18800	0.03253403	0.15183226	0.11330017
BP	GO:0097305	onse to alco	235/18800	0.03312883	0.15183226	0.11330017
BP	GO:0033673	ulation of ki	236/18800	0.03338743	0.15183226	0.11330017
BP	GO:0001835	stocyst hatch	28/18800	0.03371933	0.15183226	0.11330017
BP	GO:0032438	osome organ	28/18800	0.03371933	0.15183226	0.11330017
BP	GO:0035188	hatching	28/18800	0.03371933	0.15183226	0.11330017
BP	GO:0042983	r protein bio	28/18800	0.03371933	0.15183226	0.11330017
BP	GO:0042984	ecursor prot	28/18800	0.03371933	0.15183226	0.11330017
BP	GO:0060544	of necropto	28/18800	0.03371933	0.15183226	0.11330017
BP	GO:0071684	ence from pr	28/18800	0.03371933	0.15183226	0.11330017
BP	GO:2000765	f cytoplasmi	28/18800	0.03371933	0.15183226	0.11330017
BP	GO:0008209	en metabolic	29/18800	0.03490325	0.15310172	0.11424747
BP	GO:0048753	granule orga	29/18800	0.03490325	0.15310172	0.11424747
BP	GO:1902175	duced intrin	29/18800	0.03490325	0.15310172	0.11424747
BP	GO:0007188	protein-cou	244/18800	0.03548506	0.15310172	0.11424747
BP	GO:0007190	adenylate cy	30/18800	0.03608578	0.15310172	0.11424747

BP	GO:0061037	ion of cartila	30/18800	0.03608578	0.15310172	0.11424747
BP	GO:1903579	ion of ATP m	30/18800	0.03608578	0.15310172	0.11424747
BP	GO:0060047	art contracti	247/18800	0.03628475	0.15310172	0.11424747
BP	GO:0015833	ptide transp	249/18800	0.0368218	0.15310172	0.11424747
BP	GO:0034612	o tumor necr	249/18800	0.0368218	0.15310172	0.11424747
BP	GO:0002862	mmatory res	31/18800	0.03726692	0.15310172	0.11424747
BP	GO:0010664	triated musc	31/18800	0.03726692	0.15310172	0.11424747
BP	GO:0033137	f peptidyl-se	31/18800	0.03726692	0.15310172	0.11424747
BP	GO:0035767	elial cell cher	31/18800	0.03726692	0.15310172	0.11424747
BP	GO:0045987	of smooth	31/18800	0.03726692	0.15310172	0.11424747
BP	GO:0046825	rotein expor	31/18800	0.03726692	0.15310172	0.11424747
BP	GO:0062098	grammed n	31/18800	0.03726692	0.15310172	0.11424747
BP	GO:0045927	regulation o	256/18800	0.03872589	0.15827965	0.11811134
BP	GO:0003015	heart process	257/18800	0.03900097	0.15859069	0.11834345
BP	GO:0009648	otoperiodis	33/18800	0.03962505	0.15869932	0.11842451
BP	GO:0010765	ion of sodiu	33/18800	0.03962505	0.15869932	0.11842451
BP	GO:0045940	n of steroid	33/18800	0.03962505	0.15869932	0.11842451
BP	GO:0061448	e tissue dev	260/18800	0.03983079	0.1587257	0.11844419
BP	GO:0032412	nsmembrane	263/18800	0.04066741	0.16098628	0.12013108
BP	GO:0033238	ular amine n	34/18800	0.04080204	0.16098628	0.12013108
BP	GO:0019228	al action po	35/18800	0.04197766	0.16320093	0.1217837
BP	GO:0044060	of endocrir	35/18800	0.04197766	0.16320093	0.1217837
BP	GO:0048048	ic eye morph	35/18800	0.04197766	0.16320093	0.1217837
BP	GO:0051348	ation of tran	270/18800	0.04264564	0.16431081	0.12261191
BP	GO:0032350	ormone me	36/18800	0.04315189	0.16431081	0.12261191
BP	GO:0050931	t cell differer	36/18800	0.04315189	0.16431081	0.12261191
BP	GO:0071312	response to	36/18800	0.04315189	0.16431081	0.12261191
BP	GO:0022898	membrane t	273/18800	0.04350452	0.16431081	0.12261191
BP	GO:0008207	ormone met	37/18800	0.04432475	0.16431081	0.12261191
BP	GO:0032094	sponse to fo	37/18800	0.04432475	0.16431081	0.12261191
BP	GO:0038179	hin signaling	37/18800	0.04432475	0.16431081	0.12261191
BP	GO:0045762	n of adenyla	37/18800	0.04432475	0.16431081	0.12261191
BP	GO:0071276	ponse to cac	37/18800	0.04432475	0.16431081	0.12261191
BP	GO:0035886	mooth musc	38/18800	0.04549623	0.16721297	0.12477756
BP	GO:0046879	mone secret	281/18800	0.04582677	0.16721297	0.12477756
BP	GO:0042307	of protein in	39/18800	0.04666634	0.16721297	0.12477756
BP	GO:0042755	ating behavi	39/18800	0.04666634	0.16721297	0.12477756
BP	GO:0045823	ation of hea	39/18800	0.04666634	0.16721297	0.12477756
BP	GO:0071470	ponse to osr	39/18800	0.04666634	0.16721297	0.12477756
BP	GO:0090278	of peptide	39/18800	0.04666634	0.16721297	0.12477756
BP	GO:0001666	onse to hyp	286/18800	0.0473014	0.16721297	0.12477756
BP	GO:0042886	nide transpo	287/18800	0.04759845	0.16721297	0.12477756
BP	GO:0002792	lation of pep	40/18800	0.04783508	0.16721297	0.12477756
BP	GO:0071392	nse to estra	40/18800	0.04783508	0.16721297	0.12477756
BP	GO:0097009	rgy homeost	40/18800	0.04783508	0.16721297	0.12477756
BP	GO:1903524	lation of blo	40/18800	0.04783508	0.16721297	0.12477756

BP	GO:0009914	mone transp	290/18800	0.04849377	0.16834029	0.12561879
BP	GO:0046688	nse to coppe	41/18800	0.04900244	0.16834029	0.12561879
BP	GO:0071548	e to dexame	41/18800	0.04900244	0.16834029	0.12561879
BP	GO:0120178	one biosyntl	41/18800	0.04900244	0.16834029	0.12561879
CC	GO:0043679	xon terminu	113/19594	8.53E-06	0.0006196	0.00043228
CC	GO:0044306	projection te	129/19594	1.44E-05	0.0006196	0.00043228
CC	GO:0150034	distal axon	270/19594	0.00025399	0.00728094	0.00507973
CC	GO:0031045	ise core gran	26/19594	0.00042108	0.0090532	0.00631618
CC	GO:0043195	rminal bouto	48/19594	0.0014387	0.02474571	0.01726445
CC	GO:0098793	presynapse	492/19594	0.00237972	0.03410937	0.02379723
CC	GO:0030665	ated vesicle	111/19594	0.00744506	0.09041764	0.06308207
CC	GO:0030018	Z disc	127/19594	0.00964696	0.09041764	0.06308207
CC	GO:0031674	I band	139/19594	0.01146596	0.09041764	0.06308207
CC	GO:0043204	perikaryon	153/19594	0.0137638	0.09041764	0.06308207
CC	GO:0044292	ndrite termir	12/19594	0.01399926	0.09041764	0.06308207
CC	GO:0060198	n-sculpted v	12/19594	0.01399926	0.09041764	0.06308207
CC	GO:0098992	al dense core	13/19594	0.01515737	0.09041764	0.06308207
CC	GO:0030662	l vesicle mem	176/19594	0.01793392	0.09041764	0.06308207
CC	GO:0043025	ronal cell bo	482/19594	0.01817605	0.09041764	0.06308207
CC	GO:0005767	ndary lysoso	17/19594	0.01977679	0.09041764	0.06308207
CC	GO:0033162	osome mem	18/19594	0.02092841	0.09041764	0.06308207
CC	GO:0045009	chitosome	18/19594	0.02092841	0.09041764	0.06308207
CC	GO:0090741	t granule me	18/19594	0.02092841	0.09041764	0.06308207
CC	GO:0030136	rin-coated ve	192/19594	0.02111252	0.09041764	0.06308207
CC	GO:0060077	ibitory synap	19/19594	0.02207873	0.09041764	0.06308207
CC	GO:0030017	sarcomere	209/19594	0.02472827	0.09666505	0.06744073
CC	GO:0030016	myofibril	228/19594	0.02904792	0.10861395	0.07577718
CC	GO:0043292	ontractile fib	238/19594	0.03143503	0.11136514	0.07769661
CC	GO:0030285	t of synaptic	28/19594	0.03237359	0.11136514	0.07769661
CC	GO:0101031	perone comp	34/19594	0.03917915	0.12839146	0.08957544
CC	GO:0016235	aggresome	35/19594	0.04030895	0.12839146	0.08957544
CC	GO:0030135	oated vesicle	290/19594	0.04502684	0.13623263	0.09504602
CC	GO:0098563	t of synaptic	40/19594	0.04593891	0.13623263	0.09504602
MF	GO:0071855	tide recepto	36/18410	0.00091661	0.07882814	0.05596123
MF	GO:0048018	tor ligand ac	489/18410	0.00291479	0.08667297	0.06153038
MF	GO:0030546	eceptor activa	496/18410	0.00306798	0.08667297	0.06153038
MF	GO:0042562	rmone bindi	87/18410	0.00523641	0.08667297	0.06153038
MF	GO:0001664	oupled recep	288/18410	0.00532162	0.08667297	0.06153038
MF	GO:0005179	rmone activi	122/18410	0.01006148	0.08667297	0.06153038
MF	GO:0008190	itiation facto	10/18410	0.01242623	0.08667297	0.06153038
MF	GO:0050786	receptor bir	10/18410	0.01242623	0.08667297	0.06153038
MF	GO:0099106	nel regulato	138/18410	0.01273115	0.08667297	0.06153038
MF	GO:0016247	el regulator a	143/18410	0.01362239	0.08667297	0.06153038
MF	GO:0000774	ide exchang	11/18410	0.0136607	0.08667297	0.06153038
MF	GO:0008528	led peptide r	148/18410	0.01454016	0.08667297	0.06153038
MF	GO:0035325	e receptor b	12/18410	0.01489369	0.08667297	0.06153038

MF	GO:0001653	de receptor a	154/18410	0.01567604	0.08667297	0.06153038
MF	GO:0015355	xylate transm	13/18410	0.0161252	0.08667297	0.06153038
MF	GO:0060590	e regulator a	13/18410	0.0161252	0.08667297	0.06153038
MF	GO:0008083	vth factor act	162/18410	0.01724825	0.08725585	0.06194417
MF	GO:0036041	ain fatty acid	15/18410	0.01858382	0.08878936	0.06303284
MF	GO:0140597	n carrier chap	18/18410	0.02226073	0.10075909	0.07153032
MF	GO:0005416	cation sympo	22/18410	0.02714278	0.11671397	0.08285692
MF	GO:0015295	oton symport	25/18410	0.03078901	0.12608834	0.08951192
MF	GO:0005184	otide hormor	30/18410	0.03683703	0.13379118	0.09498027
MF	GO:0071889	-3 protein bi	30/18410	0.03683703	0.13379118	0.09498027
MF	GO:0005154	wth factor re	32/18410	0.03924612	0.13379118	0.09498027
MF	GO:0031369	initiation fac	32/18410	0.03924612	0.13379118	0.09498027
MF	GO:0030291	eonine kinas	33/18410	0.0404485	0.13379118	0.09498027
MF	GO:0045505	rmediate cha	36/18410	0.04404701	0.14029787	0.09959947
MF	GO:0017080	annel regula	40/18410	0.04882494	0.14534942	0.10318564

Count
2
2
3
4
2
3
3
2
3
2
3
3
2
3
3
2
3
2
2
2
2
2
2
3
2
2
2
3
2
2
3
3
3
3
1
1
1
1
2
2
2
1
1
1

1
1
1
1
2
2
2
1
1
1
1
1
1
3
3
3
2
1
1
1
1
1
1
1
1
1
3
2
1
1
1
1
1
1
3
1
1
1
1
1
1
1
1
1
1
2

2
1
1
1
1
1
1
1
2
2
2
2
2
3
1
1
1
2
2
2
1
1
1
1
1
2
2
1
1
1
1
1
1
1
1
1
1
1
1
1
1
1
1
2
2
1

1
1
1
1
1
1
1
1
1
1
1
1
2
2
1
1
1
1
1
2
1
1
1
1
1
2
2
1
1
1
1
2
2
1
1
1
1
1
1
1
1
1
1
1
2
1

1
1
2
2
2
1
1
1
1
1
1
1
2
2
1
1
1
2
2
1
1
1
1
2
1
1
1
2
1
1
1
1
1
1
2
1
1
1
1
1
2
2
1
1
1
1

2
1
1
1
4
4
4
2
2
4
2
2
2
2
1
1
1
2
3
1
1
1
1
2
1
2
2
2
1
1
1
2
1
2
4
4
2
3
2
1
1
2
2
1
2
1

2
1
1
2
1
1
1
1
1
1
1
1
1
1
1
