# Supplementary material for: Identification of hub genes and construction of diagnostic nomogram model in schizophrenia
Source: Front Aging Neurosci. 2022 Oct 14;14:1032917. doi: 10.3389/fnagi.2022.1032917 (PMC9614240; doi:10.3389/fnagi.2022.1032917)
Supplement: Supplementary file 3 [file Data_Sheet_3.PDF]

Supplementary Table 3. DO enrichment

ID	Description	BgRatio	pvalue	p.adjust	qvalue	Count
DOID:9970	obesity	313/8007	0.00027052	0.02539207	0.01967326	5
DOID:654	overnutrition	322/8007	0.00030872	0.02539207	0.01967326	5
DOID:374	ıtrition disea	338/8007	0.00038668	0.02539207	0.01967326	5
DOID:1936	therosclerosi	344/8007	0.00403979	0.12690876	0.09832632	4
DOID:2348	tic cardiovas	345/8007	0.00408219	0.12690876	0.09832632	4
DOID:2349	rteriosclerosi	356/8007	0.00456896	0.12690876	0.09832632	4
DOID:13378	wasaki disea	54/8007	0.00504261	0.12690876	0.09832632	2
DOID:1602	ymphadeniti	58/8007	0.00579786	0.12690876	0.09832632	2
DOID:9942	ph node dise	58/8007	0.00579786	0.12690876	0.09832632	2
DOID:9352	2 diabetes m	215/8007	0.00825592	0.16085081	0.12462393	3
DOID:3908	ll cell lung c	431/8007	0.00898152	0.16085081	0.12462393	4
DOID:4905	creatic carcin	237/8007	0.01077906	0.17695616	0.13710202	3
DOID:3717	c adenocarci	94/8007	0.01470315	0.18068126	0.13998815	2
DOID:75	atic system c	101/8007	0.01684974	0.18068126	0.13998815	2
DOID:13938	amenorrhea	10/8007	0.01981482	0.18068126	0.13998815	1
DOID:5200	y tract obstru	10/8007	0.01981482	0.18068126	0.13998815	1
DOID:9008	oriatic arthri	10/8007	0.01981482	0.18068126	0.13998815	1
DOID:11400	yelonephriti	11/8007	0.02177593	0.18068126	0.13998815	1
DOID:11504	omic neuro	11/8007	0.02177593	0.18068126	0.13998815	1
DOID:2089	constipation	11/8007	0.02177593	0.18068126	0.13998815	1
DOID:2744	pyelitis	11/8007	0.02177593	0.18068126	0.13998815	1
DOID:9779	wel dysfuncti	11/8007	0.02177593	0.18068126	0.13998815	1
DOID:824	periodontitis	120/8007	0.02330412	0.18068126	0.13998815	2
DOID:8502	ous skin dise	12/8007	0.02373336	0.18068126	0.13998815	1
DOID:1793	ncreatic canc	319/8007	0.02385473	0.18068126	0.13998815	3
DOID:11202	hyperparathy	15/8007	0.02958363	0.18068126	0.13998815	1
DOID:11465	nervous syst	15/8007	0.02958363	0.18068126	0.13998815	1
DOID:3388	odontal dise	139/8007	0.0306226	0.18068126	0.13998815	2
DOID:2055	ımatic stress	17/8007	0.03346553	0.18068126	0.13998815	1
DOID:2621	ervous syste	375/8007	0.03623727	0.18068126	0.13998815	3
DOID:769	euroblastom	375/8007	0.03623727	0.18068126	0.13998815	3
DOID:5517	nach carcinc	153/8007	0.03652997	0.18068126	0.13998815	2
DOID:9206	rett's esopha	19/8007	0.03733287	0.18068126	0.13998815	1
DOID:1074	kidney failure	156/8007	0.03784984	0.18068126	0.13998815	2
DOID:299	lenocarcinon	158/8007	0.03874007	0.18068126	0.13998815	2
DOID:15	ctive system	386/8007	0.03900123	0.18068126	0.13998815	3
DOID:10883	herpangina	20/8007	0.0392611	0.18068126	0.13998815	1
DOID:4074	as adenocar	161/8007	0.04009069	0.18068126	0.13998815	2
DOID:1091	tooth disease	162/8007	0.04054494	0.18068126	0.13998815	2
DOID:13133	ELLP syndror	21/8007	0.04118571	0.18068126	0.13998815	1
DOID:3087	gingivitis	21/8007	0.04118571	0.18068126	0.13998815	1
DOID:9743	betic neuropa	21/8007	0.04118571	0.18068126	0.13998815	1
DOID:1192	ervous syste	396/8007	0.04160774	0.18068126	0.13998815	3
DOID:11123	-Schoenlein	22/8007	0.0431067	0.18068126	0.13998815	1

DOID:1557	ty reaction ty	22/8007	0.0431067	0.18068126	0.13998815	1
DOID:3369	tive neuroec	22/8007	0.0431067	0.18068126	0.13998815	1
DOID:9809	ensitivity vas	22/8007	0.0431067	0.18068126	0.13998815	1
DOID:26	ancreas disea	171/8007	0.0447224	0.18216516	0.14113785	2
DOID:3910	adenocarcin	178/8007	0.04807983	0.18216516	0.14113785	2
DOID:13515	berous sclerc	25/8007	0.04884804	0.18216516	0.14113785	1
DOID:13543	erparathyroic	25/8007	0.04884804	0.18216516	0.14113785	1
DOID:10283	rostate cance	425/8007	0.04966747	0.18216516	0.14113785	3
