# Supplementary material for: Identification of hub genes and construction of diagnostic nomogram model in schizophrenia
Source: Front Aging Neurosci. 2022 Oct 14;14:1032917. doi: 10.3389/fnagi.2022.1032917 (PMC9614240; doi:10.3389/fnagi.2022.1032917)
Supplement: Supplementary file 4 [file Data_Sheet_4.PDF]

Supplementary Table 4. GSEA analysis of DEGs

ID	Description	NES	pvalue
hsa04724	Glutamatergic synapse	-0.491146913	-1.890082745
hsa04728	Dopaminergic synapse	-0.468580391	-1.850181954
hsa04723	Retrograde endocannabinoid signaling	-0.556920588	-2.172334065
hsa04912	GnRH signaling pathway	-0.484846155	-1.819618661
hsa05032	Morphine addiction	-0.576114666	-2.134670976
hsa04727	GABAergic synapse	-0.580561699	-2.129066755
hsa05033	Nicotine addiction	-0.650557963	-2.037676379
hsa05144	Malaria	0.617909237	2.026310642
hsa04978	Mineral absorption	0.642437284	2.157849634
hsa05206	MicroRNAs in cancer	0.424901882	1.772251311
hsa04550	Signaling pathways regulating pluripotency of stem cells	0.452470988	1.838522637
hsa05205	Proteoglycans in cancer	0.370809525	1.619046838
hsa04060	Cytokine-cytokine receptor interaction	0.409424631	1.796442058
hsa01212	Fatty acid metabolism	0.603351594	1.978571743
hsa04742	Taste transduction	-0.544083055	-1.876386413
hsa05224	Breast cancer	0.396102535	1.641976035
hsa04390	Hippo signaling pathway	0.397974435	1.651337079
hsa04721	Synaptic vesicle cycle	-0.510031012	-1.789263535
hsa00532	Glycosaminoglycan biosynthesis - chondroitin sulfate / dermatan sulfate	0.723352421	1.893115969
hsa04330	Notch signaling pathway	0.507629112	1.79371765
hsa04726	Serotonergic synapse	-0.450922191	-1.708095122
hsa00071	Fatty acid degradation	0.585342556	1.855111438
hsa00601	Glycosphingolipid biosynthesis - lacto and neolacto series	-0.631458368	-1.854993542
hsa04979	Cholesterol metabolism	0.560130455	1.867553799
hsa04350	TGF-beta signaling pathway	0.430745456	1.651693126
hsa05418	Fluid shear stress and atherosclerosis	0.386481836	1.576180327

hsa00310	Lysine degradation	0.498496132	1.752603735
hsa05225	Hepatocellular carcinoma	0.366140714	1.535719856
hsa04713	Circadian entrainment	-0.43969616	-1.665570868
hsa04950	Maturity onset diabetes of the young	0.666117766	1.8110009
hsa05217	Basal cell carcinoma	0.49091219	1.73464807
hsa04929	GnRH secretion	-0.478130771	-1.691912816
hsa00260	Glycine, serine and threonine metabolism	0.538169952	1.756554921
hsa04925	Aldosterone synthesis and secretion	-0.426295429	-1.614808844
hsa04512	ECM-receptor interaction	0.418940949	1.600583107
hsa04210	Apoptosis	0.379055252	1.526492918
hsa04392	Hippo signaling pathway - multiple species	0.569126375	1.751515281
hsa04657	IL-17 signaling pathway	0.428757653	1.602372498
hsa04971	Gastric acid secretion	-0.450498613	-1.606026733
hsa05412	Arrhythmogenic right ventricular cardiomyopathy	0.432666967	1.601530542
hsa04750	Inflammatory mediator regulation of TRP channels	-0.413671808	-1.552502653
hsa04720	Long-term potentiation	-0.445676062	-1.584215154
hsa04610	Complement and coagulation cascades	0.461221996	1.649677946
hsa04936	Alcoholic liver disease	0.371202165	1.500534845
hsa04114	Oocyte meiosis	-0.40618123	-1.548259733
hsa04024	cAMP signaling pathway	-0.338400907	-1.438444099
hsa03320	PPAR signaling pathway	0.448228762	1.589669935
hsa04914	Progesterone-mediated oocyte maturation	-0.413534807	-1.508915647
hsa04064	NF-kappa B signaling pathway	0.382867996	1.489825679
hsa05226	Gastric cancer	0.346437733	1.431182395
hsa04260	Cardiac muscle contraction	-0.456087478	-1.552892462
hsa00270	Cysteine and methionine metabolism	0.483284815	1.593780235
hsa05220	Chronic myeloid leukemia	0.40295934	1.511715364
hsa05219	Bladder cancer	0.481496399	1.587882386

hsa04933	AGE-RAGE signaling pathway in diabetic complications	0.36981153	1.456110158
hsa00510	N-Glycan biosynthesis	-0.519866302	-1.600396051
hsa04725	Cholinergic synapse	-0.376814494	-1.452743492
hsa04935	Growth hormone synthesis, secretion and action	-0.362580193	-1.426550847
hsa04921	Oxytocin signaling pathway	-0.35180975	-1.411899963
hsa05130	Pathogenic Escherichia coli infection	0.329089201	1.396734415
hsa05169	Epstein-Barr virus infection	0.327498922	1.389984887
hsa00120	Primary bile acid biosynthesis	0.687435793	1.636763143
hsa05167	Kaposi sarcoma-associated herpesvirus infection	0.321313018	1.367932834
hsa04972	Pancreatic secretion	-0.398951757	-1.453069514
hsa04722	Neurotrophin signaling pathway	-0.35950135	-1.409305422
hsa04911	Insulin secretion	-0.391733947	-1.438674671
hsa00561	Glycerolipid metabolism	0.451640658	1.505833543
hsa00982	Drug metabolism - cytochrome P450	0.455685696	1.494330752
hsa05014	Amyotrophic lateral sclerosis	-0.306723735	-1.316610748
hsa05171	Coronavirus disease - COVID-19	0.321758826	1.33901695
hsa05323	Rheumatoid arthritis	0.388794736	1.427491352
hsa05222	Small cell lung cancer	0.356790385	1.388351816
hsa04261	Adrenergic signaling in cardiomyocytes	-0.337681451	-1.350840299
hsa04668	TNF signaling pathway	0.355891312	1.384853321
hsa05034	Alcoholism	-0.372200494	-1.392056646
hsa04066	HIF-1 signaling pathway	0.354383928	1.378987749
hsa00380	Tryptophan metabolism	0.470848203	1.492247363
hsa00512	Mucin type O-glycan biosynthesis	-0.491995542	-1.48604619
hsa05016	Huntington disease	-0.311432295	-1.301428673
hsa00640	Propanoate metabolism	0.495818358	1.494385474
hsa05212	Pancreatic cancer	0.362940556	1.376919563
hsa05012	Parkinson disease	-0.322780036	-1.312015199

hsa04630	JAK-STAT signaling pathway	0.32503123	1.323670497
hsa04614	Renin-angiotensin system	0.591756306	1.519525736
