# Supplementary figures and images for: Identification of hub genes and construction of diagnostic nomogram model in schizophrenia
Source: Front Aging Neurosci. 2022 Oct 14;14:1032917. doi: 10.3389/fnagi.2022.1032917 (PMC9614240; doi:10.3389/fnagi.2022.1032917)

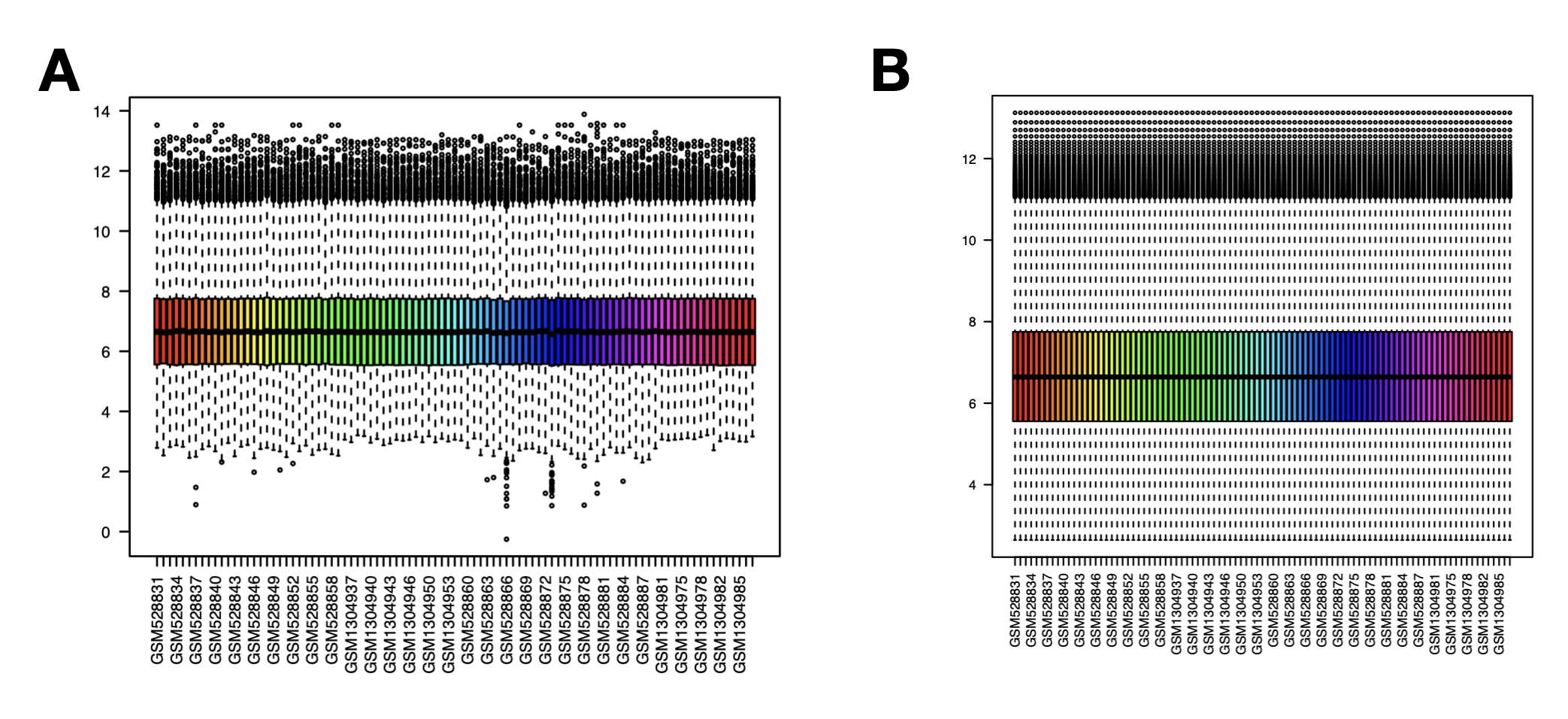

Supplement: Supplementary file 5 [file Image_1.PNG]

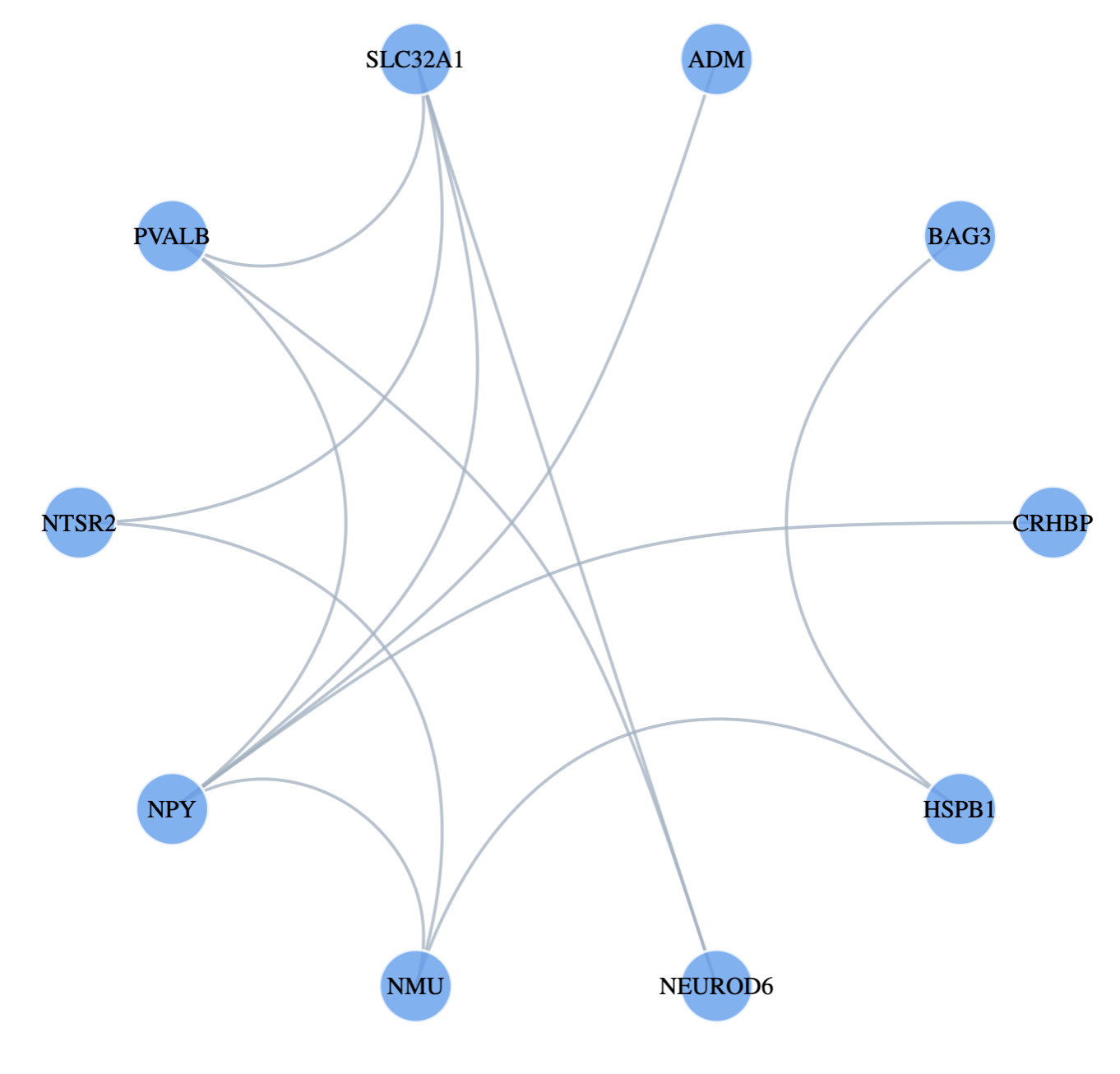

Supplement: Supplementary file 6 [file Image_2.PNG]

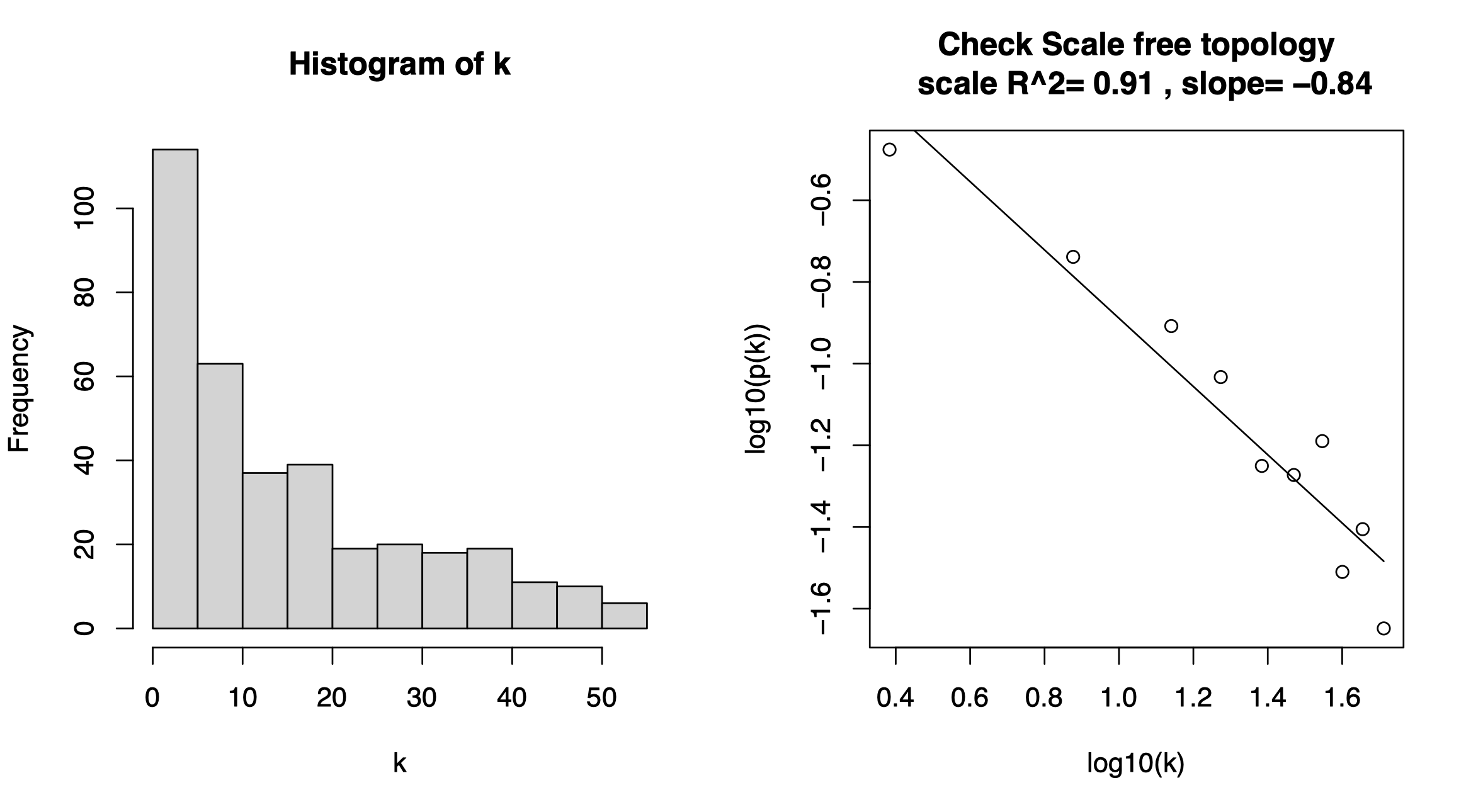

Supplement: Supplementary file 7 [file Image_3.PNG]

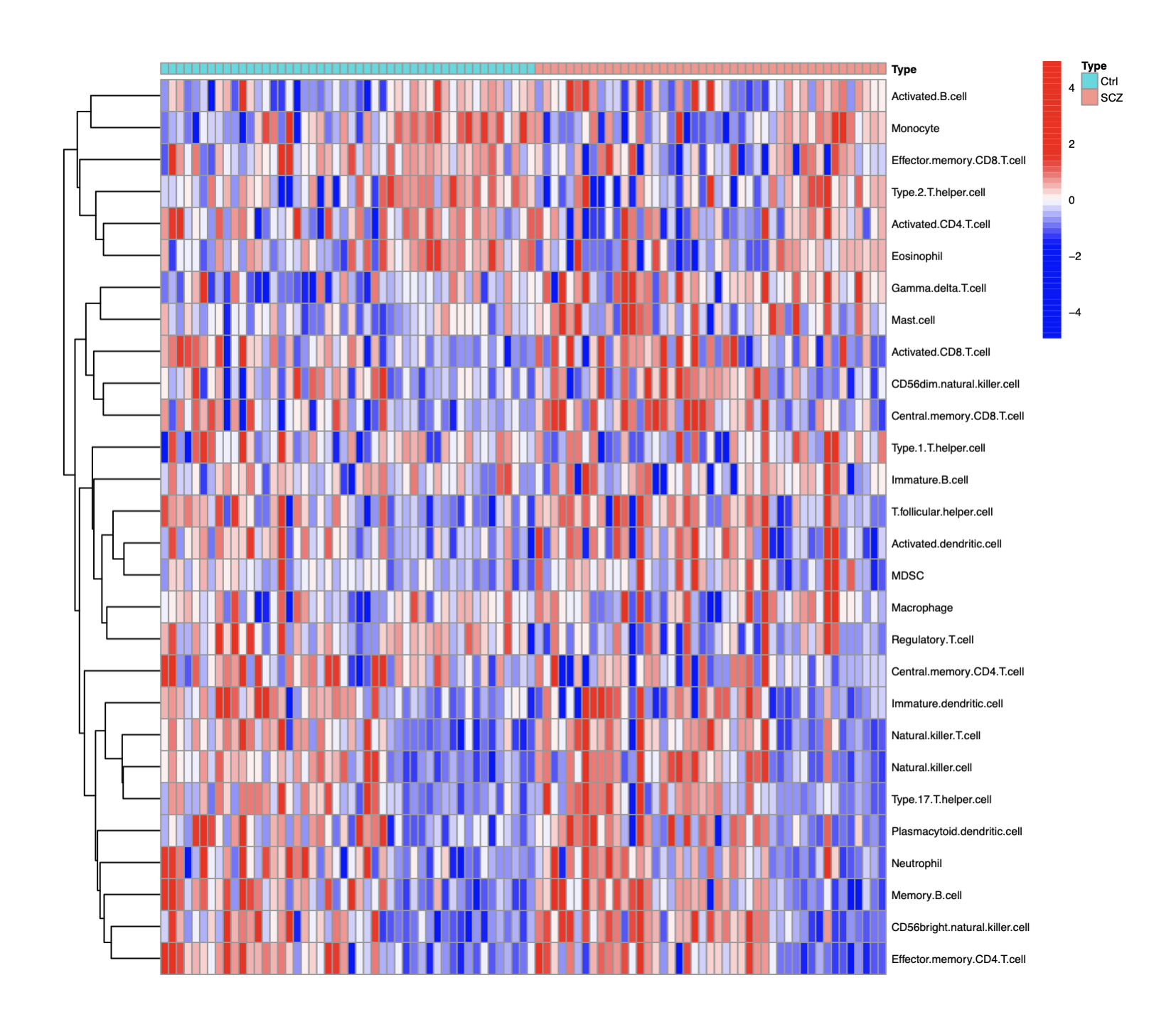

Supplement: Supplementary file 8 [file Image_4.PNG]
